# Management practices and human–horse relationships are associated with horse personality traits

**DOI:** 10.1098/rsos.260116

**Published:** 2026-09-09

**Authors:** Alisa Viitanen, Aada Ståhl, Milla K. Salonen, Océane Liehrmann

**Affiliations:** ^1^ Department of Biology, University of Turku, Turku, Finland; ^2^ Department of Veterinary Biosciences, University of Helsinki, Helsinki, Finland; ^3^ Department of Medical and Clinical Genetics, University of Helsinki, Helsinki, Finland; ^4^ Folkhälsan Research Center, Helsinki, Finland; ^5^ Department of Biosystems and Technology, Swedish University of Agricultural Sciences, Alnarp, Sweden

**Keywords:** riding frequency, horse welfare, horse bit, equestrianism, human–animal relationship

## Abstract

The human–horse relationship has evolved through centuries of domestication, shaping how horses are managed and interact with people. Horses show interindividual behavioural differences that constitute personality, with implications for welfare, management and human–horse interactions. Although equine personality has a genetic component, environmental and relational factors may influence its expression, yet effects of everyday management practices remain poorly understood. This study examined associations between owner-rated horse personality traits and human-controlled environmental and relationship parameters. Owners completed an online questionnaire (2767 horses) assessing horse personality and collecting data on housing conditions, riding, groundwork frequency, quality time, relationship duration, riders and ownership history, and headgear use. Analyses controlled for intrinsic horse characteristics (age, sex and breed). We identified four personality dimensions: human sociability, horse sociability, attentiveness and neuroticism. Human sociability was positively associated with age, quality time and relationship stability, and negatively associated with multiple owner changes. Horse sociability was associated with housing, with lower scores in singly housed horses. Attentiveness increased with age and riding frequency, while neuroticism was lower in more frequently ridden horses. Overall, personality ratings were associated with intrinsic characteristics and management and relationship factors, highlighting the importance of the human-shaped environment for equine personality and welfare.

## 1. Introduction

The relationship between humans and horses is complex owing to the versatile uses of horses and the evolution of their status throughout history. This relationship began around 2200 BC in the Western Eurasian steppes with the domestication of *Equus caballus* for transportation [1]. The rise of horseback riding and the use of horses for transportation was a pivotal development in human history, enabling exploration, territorial expansion, trade growth and significant improvements in agricultural efficiency [1]. Over recent decades, horses in Western societies have shifted from primarily utilitarian roles to being valued as companions and leisure partners, with recreational, social and sporting uses now dominant, and horses commonly viewed as companion animals rather than purely working stock [2].

Horses are prey animals [3], who naturally form stable social groups called harems, composed of mares and a few stallions [4]. Females often remain in the same group for life, forming strong bonds [5]. In the wild, horses graze for 12–18 hours daily and cover 9–16 km [6,7], making them highly active animals. Since their domestication, horses have gone through changes in their behaviour, becoming more curious and more social and less agonistic towards humans [8]. While becoming accustomed to human-controlled environments, horses have experienced new conditions, including changes in housing, herd size, activities, riding gear and human interactions. Nowadays, horse housing varies in space and social access. Some horses are kept alone in individual stalls, while others may live in pasture within a herd [9,10]. Outdoor group housing mimics natural conditions more closely [11] and allows more social and movement-based behaviours [12,13]. In contrast, isolation limits social contact and tends to increase alertness, disrupt sleep and be associated with nervousness and aggression [5,14].

Horses exhibit pronounced and consistent interindividual differences in personality and behaviour, a phenomenon that has been widely documented across experimental, observational and large-scale studies [15]. Traits, such as fearfulness, reactivity to humans, gregariousness, learning style and emotional responsiveness, show temporal stability and consistency across contexts, meeting the criteria for animal personality [16]. These individual differences are not merely descriptive but have important implications for welfare, performance, injury risk and the quality of horse–human relationships, making personality a key factor in equine management and training [17,18]. A growing body of evidence indicates that equine personality traits have a substantial genetic and hereditary component. Systematic reviews have reported moderate heritability estimates for several personality dimensions and identified consistent breed differences, as well as candidate genes and polymorphisms associated with traits such as curiosity, vigilance, tractability and emotional reactivity [15,19–21]. However, genetic predispositions do not act in isolation; rather, they interact with environmental conditions to shape behavioural expression throughout the horse's life. Housing conditions, type of work or discipline, early social experiences with the dam, training methods and the quality and consistency of human handling have all been shown to influence horses’ emotional responses, learning abilities and reactions to social or physical challenges [15,22]. Despite this extensive body of research, important gaps remain in our understanding of how these factors interact under everyday conditions. Existing literature often focuses on isolated environmental variables or specific horse–human interactions in controlled settings. While daily handling, management and rider numbers affect a horse's emotional reactivity and welfare, with these effects varying according to individual personality traits [16,18], we still know little about how relationship histories and daily care influence personality expression across large, diverse populations. Addressing this gap is essential to better guide individual management, training and welfare decisions.

Accordingly, this study aims to deepen our understanding of the interplay between horse interindividual variation in personality and the human-shaped environment in which they interact and build their relationship. To investigate this, we conducted an online questionnaire-based study. The questionnaire is divided into two main sections. The first part assesses human-controlled factors. These include management practices (such as turnout and herd dynamics), training and equipment usage, and relationship history, which captures the duration, frequency and nature of horse–owner interactions, while the second part focuses on horse personality. Finally, to ensure a thorough analysis, we controlled for intrinsic horse factors, such as age, sex and breed-related parameters, while also accounting for raters’ information as random factors. These parameters were, therefore, examined in relation to personality ratings in the present study.

## 2. Material and methods

### 2.1. Survey

The questionnaire used for this study consisted of two sections: a background information section and a horse personality assessment. It was distributed worldwide between June 2023 and February 2024 as an online survey and was available in Finnish, French and English.

The first section used 25 questions to assess respondents’ interactions with their horses, focusing on the frequency of activities such as riding, groundwork and spending quality time together. It also collected details about horse housing, including herd size and the amount of time horses spend in pasture per year. Additionally, it asked about the types of headgear used, categorizing headgear as bitless only, bit-only or a combination of them if the owners use both options. Information about the horses, such as their sex, age and breed, was gathered, along with background details about the owners, including their biological sex, age and country of residence. Lastly, the questionnaire addressed the duration of the owner–horse relationship and the number of horses owned. All details and sample sizes are reported in tables 1 and 2.

**Table 1.** Definitions and sample sizes of the different predictors.

predictors			sample size	additional details	definitions
**riding frequency**	ordinal factor	non-broken or retired	376	Non-ridden horses younger than 5 years old were considered not broken yet. Non-ridden horses from 18 years old were considered retired.	how often the horse owner rides/drives their horse
		never	208	horses not ridden for other reasons than owing to their age	
		rarely	385		
		once or twice a month	267		
		once or twice a week	919		
		4–7 times a week	612		
**groundwork frequency**	ordinal factor	never	178		how often the horse owner trains their horses from the ground (not on the horse)
		rarely	408		
		once or twice a month	539		
		once or twice a week	1273		
		4–7 times a week	369		
**quality time frequency**	ordinal factor	never/rarely	121	owing to the small number of ‘never’ responses here, it was grouped with ‘rarely’	how often the respondents spend time with their horse without asking anything from them (e.g. just being with the horse in the stall, the paddock or the pasture, giving them scratches or treats or just doing nothing at all)
		once or twice a month	188		
		once or twice a week	834		
		4–7 times a week	1624		
**headgear**	ordinal factor	horse not ridden/trained	518		the type of headgear used during riding or training; respondents could choose multiple options and were classified based on whether they selected only bitless headgear, only headgear with bits or a combination of both
		bitless only	78	the owner only uses bitless options when training or riding the horse (e.g. neck rope, rope, classic halter, bitless bridles)	
		bitless and bit	784	the owner uses both bitless and bit headgear when training/riding the horse	
		bit only	1387	the owner only uses headgear with a bit when training/riding the horse	
**relationship length**	ordinal factor	0–1 year	286		the duration, in years, that the respondent interacted with their horse
		2–5 years	925		
		6–10 years	788		
		more than 10 years	768		
**previous owners**	factor	horse is with breeder	314	the respondent and owner of the horse is also its breeder	the number of ownership changes the horse has had, varying from living with the breeder to multiple ownership changes
		horse is bought from the breeder	968		
		multiple	1485		
**riders’ context**	not ridden		522	the horse is not ridden	the number and the type of riders the horse has—whether it is ridden with a small circle of familiar riders or with several, possibly unfamiliar, riders
	exclusive rider		1492	the horse has one rider	
	friends/family		467	friends and family also ride the horse	
	shared with others		286	the horse is ridden by several people, outside of friends and family	
**herd size**	factor	alone	407	the horse lives alone	the number of other horses the horse lives with
		dyad	852	the horse lives with one other horse	
		herd	1508	the horse lives with several other horses	
**horse sex**	factor	mare	1203	female	sex of the horse: males are categorized according to their sterilization status; females are usually not sterilized.
		gelded	1458	sterilized male	
		stallion	106	non-sterilized male	
**pasture time**	factor	never	313	never goes to pasture	the amount of time the horse spends at pasture (field greater than 1 ha with possibility to graze)
		1–5 m	719	between 1 and 5 months per year	
		6–11 m	316	between 6 and 11 months per year	
		always	1419	is always at pasture	
**horse age**	continuous		2767	min = 0.5, max = 40, mean = 13.4 and s.d. = 6.8	age of the horse in years
**breed**	draught horse		201	e.g. Percheron, Irish cob	horse breeds divided into 5 different categories based on their genetic background [28,29];
	Nordic		464	e.g. Finnish coldblood, fjord and Islandic	
	pony		186	e.g. Welsh ponies, Shetlands	
	warm-blooded		1589	e.g. American trotter, Anglo-Arabian	
	unknown		327	e.g. cross-breeds of breeds from different categories and horses of unknown origins	details are provided in the supplementary material

**Table 2.** Definitions and sample sizes of the random factors.

**random factors**			sample size		
**location**	factor	Central Europe	1234	France, Belgium, Switzerland, Germany, Austria, Monaco, Spain, Italy, Luxembourg and The Netherlands	geographical location of the respondent
		Fennoscandia	936	Norway, Sweden, Denmark and Finland	
		Ireland and the UK	227	Ireland and Great Britain	
		North America	263	Canada and the USA	
		Oceania and SA	107	Australia, New Zealand, Pacific Ocean islands and South Africa	
**human age**	ordinal factor	18–24	160		human age in categories; respondents under 18 years old were not allowed to answer the survey
		25–34	603		
		35–44	715		
		45–54	643		
		55–64	477		
		65 and over	169		
**human sex**	factor	female	2616		human biological sex
		other	151	men, intersex and respondents who did not wish to answer	
**human ID**	factor		2257	number of horses respondents filled in the survey for mean = 1, s.d. = 0.59, min = 1 and max = 8	the anonymized ID number given to the respondent as they were allowed to fill in the survey for several horses

The second section of the questionnaire consisted of a horse personality questionnaire featuring 52 items, adapted from previous studies that assessed horse personality [23–26] and additional items to provide a greater focus on the human–horse interactions. Each item was presented as a statement (e.g. ‘My horse seeks physical closeness with known humans’) to which respondents were required to indicate their level of agreement with each adjective using a five-point Likert scale: ‘strongly disagree’, ‘disagree’, ‘neutral’, ‘agree’, ‘strongly agree’, plus the option to respond ‘I don't know’. The personality questionnaire is available as supplementary material in the data repository [27].

### 2.2. Participants

A number of 2257 horse owners completed the survey, resulting in a total of 2767 responses, as horse owners were allowed to fill out the questionnaire for as many horses as they wished (mean = 1, s.d. = 0.59, min = 1 and max = 8). The survey was available in English, Finnish and French, and we used a multimodal recruitment and snowball sampling method through a variety of channels. It included targeted social media groups focused on equine topics, both online and printed equine press, specialized forums and direct outreach to horse stables (randomly picked from Google Maps) in countries where English, Finnish or French is spoken. While recruitment was targeted in these regions, the survey was accessible to respondents from anywhere in the world. The most common age group among participants was 35–44 years. Of the respondents, 2616 were women (94.5%), and 151 identified as men, intersex or preferred not to disclose their sex (5.5%). The majority of participants were from Central Europe (44.7%), with the second most common location being Fennoscandia (34.5%). Only individuals aged 18 or older were eligible to participate in the survey. Participants could fill out the horse personality part of the questionnaire multiple times if they had more than one horse. Most horses were aged from 13 to 18 years old, and 1203 of them were mares, 106 stallions and 1458 geldings. In this study, the horse's breed was categorized into different categories based on genetic resemblance (draught horse, Nordic, pony, warm-blooded and unknown) [28,29]. The high number of cross-breeds and owners unsure of their horse's breed, along with the uneven breed distribution, prevented us from incorporating the exact breed into the analysis.

### 2.3. Horse personality factor analyses

All analyses and data handling were conducted on R v. 4.4.1, and figures were created using the *ggplot2* package [30]. An exploratory factor analysis (EFA) was performed to reduce the personality questionnaire items into a smaller number of biologically meaningful factors, which is usually done in personality research [31]. Each factor thus represents a distinct personality dimension. Before the analysis, the adjective ‘protective’ (‘My horse is protective and prevents harm or possible harm to other horses’) was removed because 21.1% of respondents answered ‘I don't know’. The data were then split into train and test sets with the package *caret* [32] to allow for an assessment of dimensionality using cross-validation with confirmatory factor analysis (CFA).

#### 2.3.1. Exploratory factor analysis

To ensure the replication and validity of the identified factor structure, a split-sample validation approach was employed. The total dataset (*N* = 2767) was randomly split into a training dataset (*N* = 1385, approx. 50% of the total sample) used for EFA and an independent validation dataset (*N* = 1382, approx. 50% of the total sample) used to confirm if the model structure holds. The suitability of the data for factor analysis was first verified using the Kaiser–Meyer–Olkin test, which yielded a value of 0.91, indicating excellent factorability. Next, the number of factors was visually assessed using a screen test, which suggested solutions with either three or six factors. Additionally, Velicer's minimum average partial (MAP) test was used to estimate the optimal number of factors, with the lowest MAP value obtained with nine factors. The decision on the number of factors was made by extracting all factor solutions ranging from 1 to 10 factors and comparing them. The comparisons were based both on the content of the factors (i.e. which variables load onto each factor) and on fit indices, particularly the Tucker–Lewis index (TLI) and the root mean square error of approximation (RMSEA). All factor analyses were conducted using polychoric correlations and mean imputation for missing values. A Heywood case occurred in factor solutions with 7–10 factors, where more than 100% of a variable's variance was explained by the factors—resulting in negative residual variance. Owing to this issue, these factor configurations were excluded from further analysis. One- and two-factor models proved difficult to interpret and were, therefore, also excluded. For the remaining models, various rotation methods were compared, and the number of items that did not load onto any factors and those with cross-loadings (loading onto two or more factors simultaneously) was calculated. From these models that best fit the data, preference was given to models offering the greatest biological relevance—that is, those best capturing meaningful behavioural variation among horses. Based on this criterion, a four-factor solution was selected, with each factor named according to the types of items it included: human sociability, attentiveness, neuroticism and horse sociability (see §3 for more details). Items that did not load onto any factors were excluded from the final analysis (*n* = 9). Based on the EFA conducted on the train data, factor scores were calculated for each horse using the ‘tenBerge’ method from the *psych* package [33], with mean imputation for missing values. These individual scores were later used for the statistical analyses. We acknowledge that treating these estimated factor scores as observed, error-free variables in the generalized linear mixed models (GLMMs) omits the inherent measurement error of the latent constructs. While this approach is standard practice when complex random-effect structures preclude the use of full structural equation modelling, it represents a limitation that can potentially inflate statistical power and lead to overly conservative standard errors. The final analysis used a Varimax rotation with four factors. Internal consistency for these factors was assessed using Cronbach's alpha and Guttman's lambda (attentiveness: alpha = 0.88, lambda = 0.90; human sociability (soc): alpha = 0.85, lambda = 0.88; neuroticism: alpha = 0.86, lambda = 0.88; horse soc: alpha = 0.78, lambda = 0.82).

#### 2.3.2. Confirmatory factor analysis

To assess the stability of the four-factor structure identified in the EFA, a CFA was applied to the test data (*N* = 1382) using the CFA function from the *lavaan* package [34]. When evaluating the CFA's four-factor structure, the standardized estimates (equivalent to loadings in the EFA) and model fit indices were examined. The relative fit indices indicated a poor fit (TLI = 0.721, comparative fit index (CFI) = 0.738), while the absolute fit index showed an acceptable fit (RMSEA = 0.078 [0.076, 0.080]). The package semTools [35] was used to calculate the RMSEA of the null model, which was 0.147, indicating that relative fit indices may not be informative. Finally, Cronbach's alpha and Guttman's lambda were calculated for this dataset to assess the reliability of survey items and to help determine whether a collection of items consistently measures the same characteristic (attentiveness: alpha = 0.89, lambda = 0.91; human soc: alpha = 0.85, lambda = 0.89; neuroticism: alpha = 0.86, lambda = 0.88; horse soc: alpha = 0.77, lambda = 0.81).

#### 2.3.3. Cross-cultural validation

CFA models were applied separately to the two data subsets of population with the most respondents: French (*N* = 1117) and Finnish (*N* = 909). This was performed as described in §2.3.2 to confirm the suitability of the four-factor model across different cultures. In this comparison, particular attention was given to standardized estimates for factors and model fit indices (French: attentiveness: alpha = 0.88, lambda = 0.90; human soc: alpha = 0.84, lambda = 0.88; neuroticism: alpha = 0.86, lambda = 0.90; horse soc: alpha = 0.78, lambda = 0.82; Finnish: attentiveness: alpha = 0.98, lambda = 0.91; human soc: alpha = 0.86, lambda = 0.90; neuroticism: alpha = 0.82, lambda = 0.85; horse soc: alpha = 0.76, lambda = 0.81).

### 2.4. Statistical analyses

GLMMs from the package *glmmTMB* [36] were used to assess how predictors, from table 1, are associated with horse scores for each personality factor (human sociability, horse sociability, neuroticism and attentiveness). The *DHARMa* package [37] was used to evaluate model dispersion, deviations, and overall fit and optimal distribution of the data (Gaussian). Human-related factors, such as ID, age group, sex and geographical area, were incorporated as random effects to account for potential socio-demographic biases. Regarding the model for the personality factor ‘horse sociability’, certain human-related parameters, such as riding frequency, groundwork frequency, quality time frequency, headgear, other riders and relationship length, were removed from the initial model, as this personality factor is based on items involving horse-on-horse interactions and not human–horse interactions. Thus, there were no biological reasons to formulate hypotheses of potential associations between horse-to-horse social behaviour and the human-related parameters explored in this study.

Multicollinearity among predictor variables was assessed using a combination of correlation analyses and variance inflation factors, *car* package [38]. Pairwise Pearson correlation matrices were computed across all predictors (with ordinal variables treated as integers and nominal factors as factor levels). Collinearity was evaluated using a two-step approach: a pairwise correlation coefficient threshold of greater than 0.70 was used to flag strong associations for closer inspection [39], while an adjusted generalized variance inflation factor (GVIF) threshold greater than 5 was considered indicative of potentially problematic multicollinearity (calculated as GVIF^1/(2*Df)^). No critical multicollinearity was detected. Only the variables ‘riding frequency’ and ‘type of headgear used’ showed a pairwise correlation (= 0.72), reflecting that owners who rode more frequently tended to predominantly use bridles with bits. However, because these variables capture conceptually distinct management and welfare dimensions (workload vs equipment type) and their corresponding adjusted GVIF values remained exceptionally low (1.45 and 1.68, respectively), well below the critical threshold of 5, both variables were retained in the analyses to avoid omitted-variable bias.

To avoid the biases and type I error inflation associated with traditional stepwise selection, an information-theoretic framework was applied. Best-fitted models (table 3) were selected based on the lowest corrected Akaike information criterion (AICc) from the candidate model set using the function dredge from the package *MuMIn* [40]. A post hoc analysis (Tukey method) was then performed using the *emmeans* package [41] to obtain the estimated marginal means (EMM) and pairwise differences between groups while controlling the family-wise error rate. Effect sizes for all mixed-effects models were quantified using standardized regression coefficients *β* and 95% confidence intervals (CIs), calculated via the *effectsize* package [38] with the method *refit*. These coefficients represent the expected change in the outcome in standard deviation (s.d.) units per one s.d. change in the predictor, allowing for a direct comparison of effect magnitudes across disparate variables, with CIs encompassing zero indicating non-meaningful effects. Percentages were calculated from pairwise ratios as (ratio − 1) × 100. This approach reflects the multiplicative nature of the model, where a negative value indicates a percentage decrease and a positive value a percentage increase. Dataset and script are provided in an open access repository [29].

**Table 3.** A summary of selected predictors for each model.

response variables (personality factors)	independent variables												random factors			
	riding freq.	groundwork freq.	quality time freq.	headgear	previous owners	riders’ context	herd size	pasture time	relationship length	horse sex	horse age^2	breed	human ID	location	human age	human sex
human sociability			✓	✓	✓	✓			✓	✓	✓		✓	✓		
attentiveness	✓		✓		✓	✓					✓		✓	✓		
neuroticism	✓				✓				✓	✓	✓	✓	✓	✓		
horse sociability	✗	✗	✗	✗		✗	✓		✗	✓	✓		✓	✓		

*Notes:* The tick marks indicate that the predictor was kept in the final model. The crosses indicate predictors removed because they were not relevant to the study.

## 3. Results

### 3.1. Personality factor loadings

Factor analysis of the questionnaire data identified **four personality dimensions** in horses, labelled **human sociability, attentiveness, neuroticism and horse sociability**. Factor loadings for all personality dimensions are presented in table 4. All four-factor scores followed a normal distribution, with means close to zero and s.d. close to one, reflecting standardized scores.

— **Human sociability** scores ranged from −3.94 to 2.55 (mean = 0.001, s.d. = 1.01).
— **Attentiveness** scores ranged from −4.59 to 2.46 (mean = −0.001, s.d. = 1.01).
— **Neuroticism** scores ranged from −2.18 to 3.61 (mean = −0.002, s.d. = 0.99).
— **Horse sociability** scores ranged from −4.32 to 2.22 (mean = −0.001, s.d. = 1.00).

**Table 4.** Loadings of personality factors from the exploratory factor analysis.

item	human sociability	attentiveness	neuroticism	horse sociability
curious with humans	**0.77**	0.01	−0.13	0.05
affectionate with strangers	**0.74**	0.02	−0.16	0.04
outreaching	**0.69**	0.31	−0.24	0.05
affectionate with familiar human	**0.67**	0.11	−0.04	0.07
playful with humans	**0.51**	−0.05	−0.13	0
easy to catch	**0.47**	0.16	−0.15	0
playful with horses	**0.44**	−0.16	−0.01	0.17
curious	**0.42**	−0.04	−0.51	0.06
kind	**0.42**	**0.54**	−0.11	0.33
sociable	**0.41**	−0.04	0.13	0.36
distrustful with horses	**−0.41**	−0.05	0.19	−0.34
distrustful with humans	**−0.56**	−0.24	0.35	−0.04
avoidant of strangers	**−0.62**	−0.18	0.3	−0.05
uninterested of humans	**−0.76**	−0.06	0.12	−0.11
caring	0.2	0.25	−0.14	**0.64**
permissive	−0.02	0.16	−0.09	**0.53**
equable	0.08	0.21	−0.34	**0.52**
submissive	−0.03	−0.02	0.14	**0.5**
popular	0.25	0.1	−0.05	**0.46**
controlling with other horses	0.06	−0.05	−0.09	**−0.48**
invasive with horses	0.07	**−0.42**	0.09	**−0.64**
aggressive with horses	−0.12	−0.19	0.13	**−0.85**
anxious	−0.14	−0.11	**0.86**	−0.06
fearful	−0.2	−0.08	**0.83**	−0.08
insecure	−0.06	−0.13	**0.8**	−0.01
excitable	−0.01	−0.26	**0.64**	−0.15
reliable	0.06	**0.59**	**−0.44**	0.17
confident	0.2	0.17	**−0.82**	0.08
respectful	−0.11	**0.65**	−0.03	0.11
responsive	0.33	**0.62**	−0.14	0.06
focused	0.07	**0.61**	−0.19	0.05
obedient	0.14	**0.59**	−0.13	0.1
consistent	0.11	**0.57**	−0.32	0.17
attentive	0.26	**0.55**	0.02	0.06
irritable	−0.19	**−0.46**	0.3	−0.33
distracted	0.11	**−0.51**	0.33	−0.05
uncooperative	−0.11	**−0.57**	0.1	−0.14
erratic	−0.16	**−0.57**	0.37	−0.19
aggressive with humans	−0.23	**−0.58**	0.08	−0.36
frustrated with constraint	−0.01	**−0.57**	0.21	−0.19
uneasy to handle	0	**−0.72**	0.08	−0.22

*Notes:* Significant loadings for each personality factor are marked in bold (over 0.4 or below −0.4).

### 3.2. Association between personality factors and environment, relationship and intrinsic parameters

For clarity and to enhance readability, only the standardized coefficients (*β*) and *p*-values of the ANOVA and post hoc comparisons of the most relevant findings are described in the main text. Full statistical results for all standardized coefficients (*β*) and their associated CIs as well as the ANOVA of the models and model-EMMs, including CIs, effect estimates, statistics ratios and *p*-values, are provided in supplementary materials, tables S1–S3. Specific post hoc pairwise comparisons mentioned in the text are expressed as percentage differences to aid interpretation.

#### 3.2.1. Human sociability

While several predictors reached statistical significance, horse age and sex produced the most pronounced shifts across the human-directed sociability spectrum.

**Figure 1.**
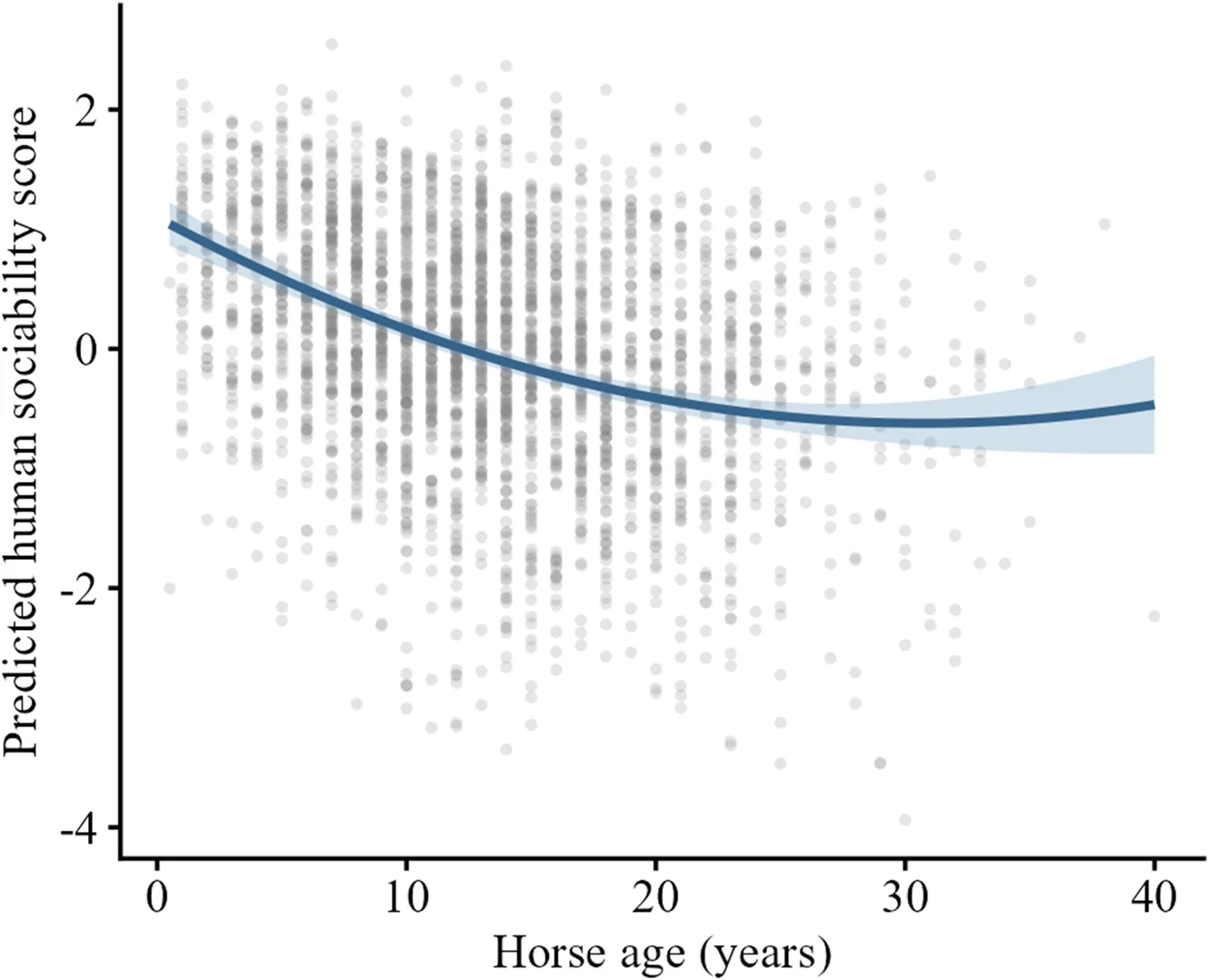
Association of human sociability score with the horse's age. The blue line demonstrates the predicted human sociability score throughout the horse's age. The light blue area represents the lower and upper 95% confidence intervals extracted from the corresponding model.

**Horse age** was the strongest predictor of sociability (*χ*² = 189.08, *p* < 0.001). The relationship was characterized by a large negative linear effect as horses matured (*β* = −20.23, *p* < 0.001), followed by a significant positive quadratic component, indicating a plateau in older horses (*β* = 5.73, *p* < 0.001) (figure 1).

**Figure 2.**
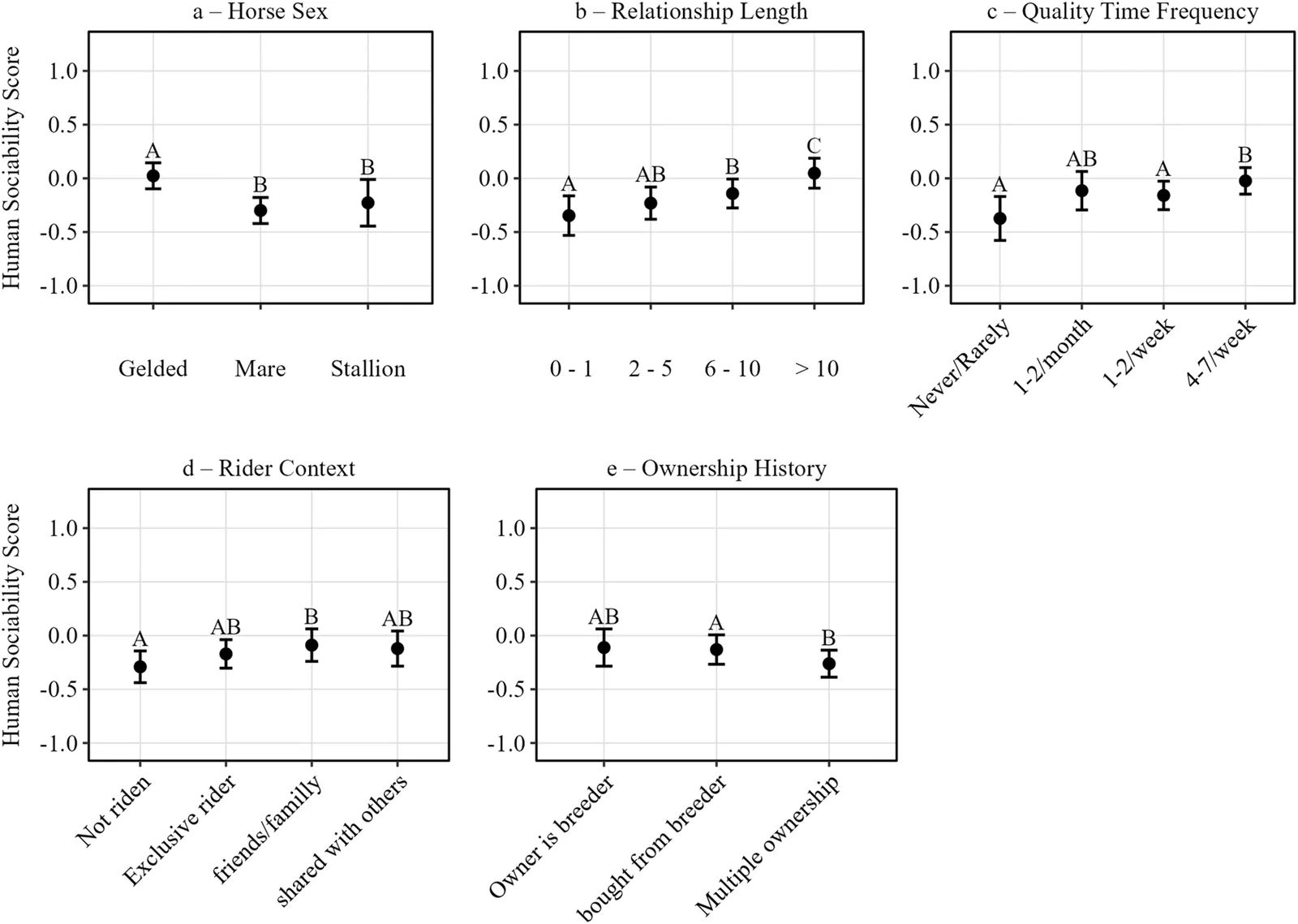
Association of human sociability score with the (a) horse sex, (b) relationship length, (c) quality time frequency, (d) rider context and (e) ownership history. Distinct letters signify statistically significant differences among the groups. The means and their associated 95% confidence intervals (error bars) were extracted from the corresponding model using the function emmeans from the ‘emmeans’ package in R.

**Horse sex** also contributed substantially to variation (*χ*² = 78.25, *p* < 0.001). Geldings exhibited the highest sociability, scoring 10.3% higher than mares (*β* = 0.32, *z* = 8.80, *p* < 0.0001) and 8.0% higher than stallions (*β* = 0.25, *z* = 2.59, *p* = 0.026), while the difference between mares and stallions was negligible and non-significant (*β* = −0.07, *z* = −0.75, *p* = 0.737) (figure 2a).

**Relationship length** showed a medium positive effect (*χ*² = 22.90, *p* < 0.001), with horses owned for more than 10 years scoring 12.3% higher than those with new owners (difference = 0.39, *z* = −4.54, *p* < 0.0001) and 8.7% higher than those with 2–5-year relationships (difference = 0.28, *z* = −4.18, *p* = 0.0002) (figure 2b).

**Frequent quality time** showed a small-to-medium positive effect (*χ*² = 23.33, *p* < 0.001). Horses receiving four to seven sessions per week scored 10.3% higher than those receiving it rarely (difference = 0.35, *z* = −3.95, *p* = 0.0005) and 4.0% higher than those receiving it one to two times per week (difference = 0.13, *z* = −3.37, *p* = 0.0042) (figure 2c).

**The social context of riding** was further associated with sociability (*χ*² = 12.90, *p* = 0.004). Horses also ridden by friends or family scored 5.9% higher than horses that were not ridden at all (*β* = 0.20, *z* = 3.38, *p* = 0.004) (figure 2d).

**Ownership history** also had a significant association (*χ*² = 9.51, *p* = 0.009), with horses purchased directly from a breeder scoring 3.4% higher than those with multiple previous owners (difference = 0.13, *z* = 2.94, *p* = 0.009) (figure 2e).

#### 3.2.2. Attentiveness

While several predictors reached statistical significance, horse age produced the most pronounced shift across this behavioural spectrum, followed by variables related to riding frequency, quality time with humans, social riding context and ownership history.

**Figure 3.**
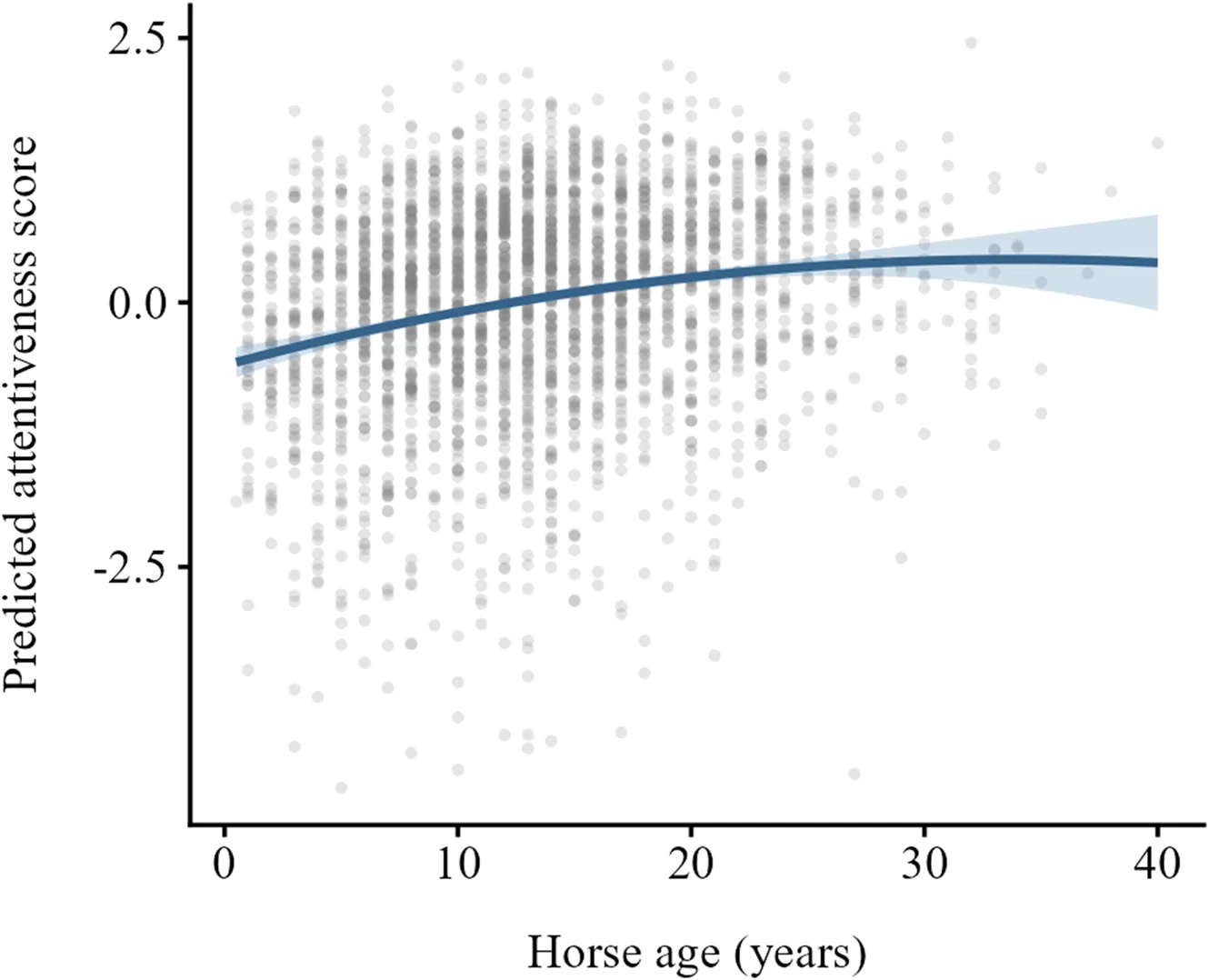
Association of attentiveness score with the horse's age. The blue line demonstrates the predicted attentiveness score throughout the horse's age. The light blue area represents the lower and upper 95% confidence intervals extracted from the corresponding model.

**Horse age** was the strongest predictor of attentiveness (*χ*² = 128.64, *p* < 0.001). With a large positive linear effect as horses matured (*β* = 11.74, *z* = 11.05, *p* < 0.001). This effect was followed by a significant negative quadratic component (*β* = −2.76, *z* = −2.33, *p* = 0.020), indicating a levelling off of attentiveness in older horses (figure 3).

**Figure 4.**
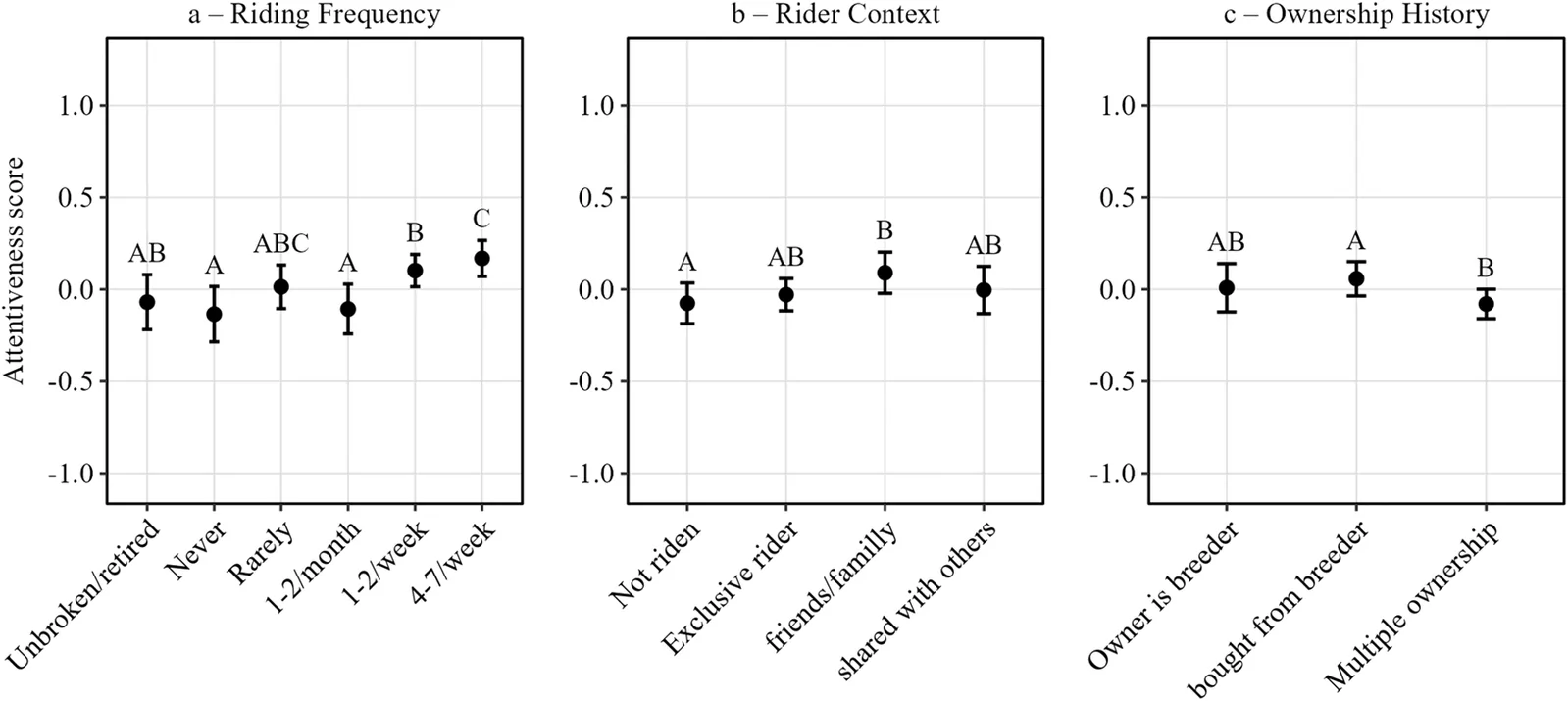
Association of attentiveness score with the (a) riding frequency, (b) rider context and (c) ownership history. Distinct letters signify statistically significant differences among the groups. The means and their associated 95% confidence intervals (error bars) were extracted from the corresponding model using the function emmeans from the ‘emmeans’ package in R.

**Riding frequency** had a significant overall effect (*χ*² = 28.82, *p* < 0.001), with a small-to-medium positive linear trend (*β* = 0.21, *z* = 3.99, *p* < 0.001). Horses ridden four to seven times per week were significantly more attentive than those never ridden (difference = 0.30, *z* = −3.84, *p* = 0.0017), corresponding to a 4.3% increase across the observed range, and than those classified as unbroken or retired (difference = 0.24, *z* = −3.16, *p* = 0.0197), corresponding to a 3.4% increase (figure 4a).

**Figure 5.**
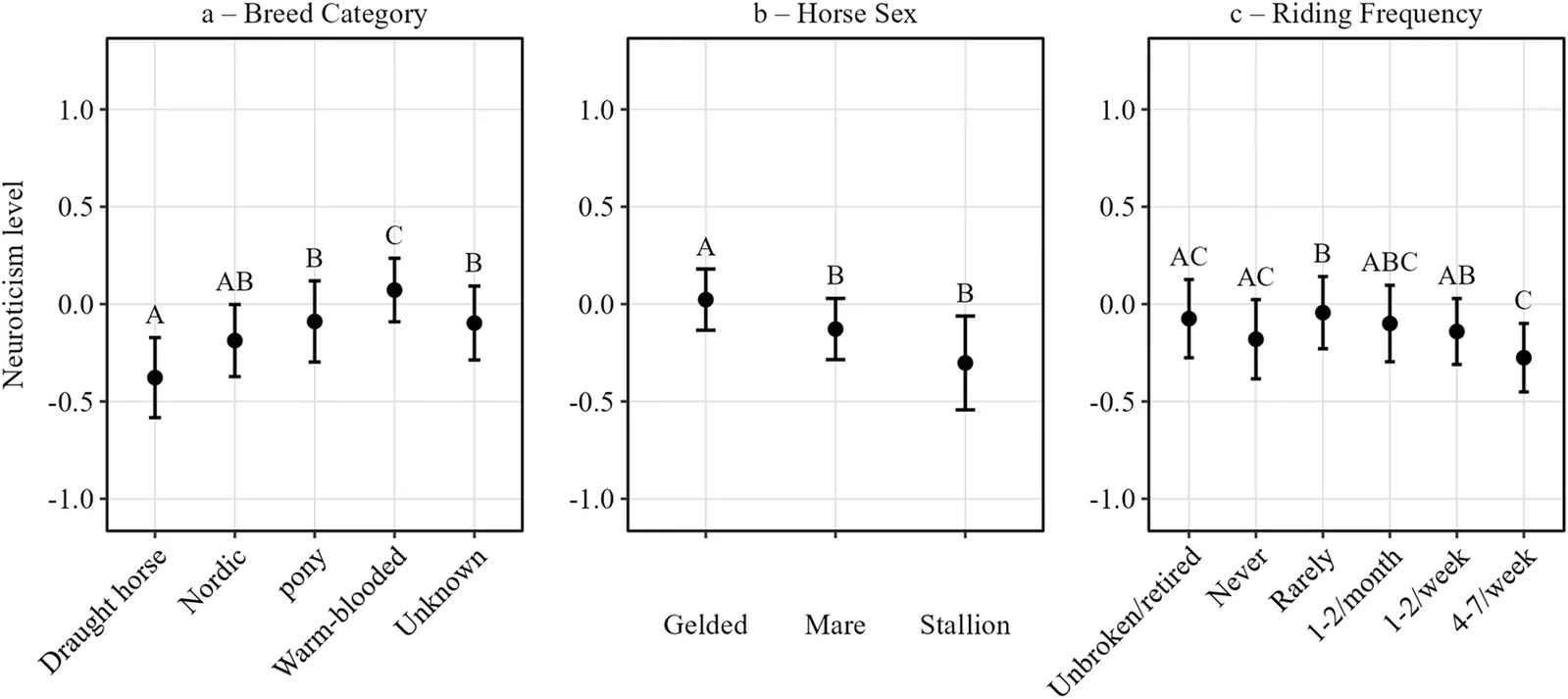
Association of neuroticism score with the (a) breed, (b) horse sex and (c) riding frequency. Distinct letters signify statistically significant differences among the groups. The means and their associated 95% confidence intervals (error bars) were extracted from the corresponding model using the function emmeans from the ‘emmeans’ package in R.

**Figure 6.**
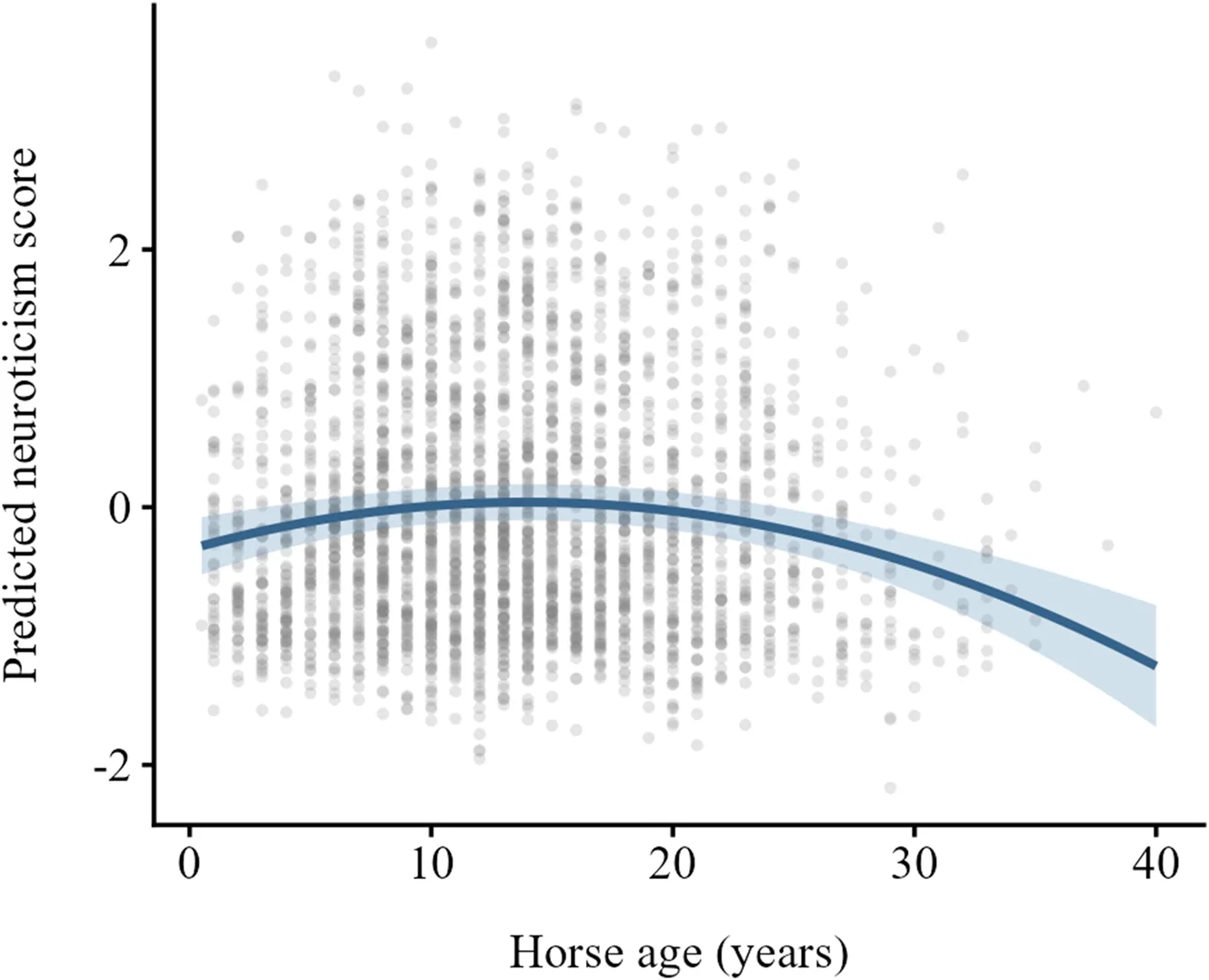
Association of neuroticism score with the horse's age. The blue line demonstrates the predicted neuroticism score throughout the horse's age. The light blue area represents the lower and upper 95% confidence intervals extracted from the corresponding model.

**Figure 7.**
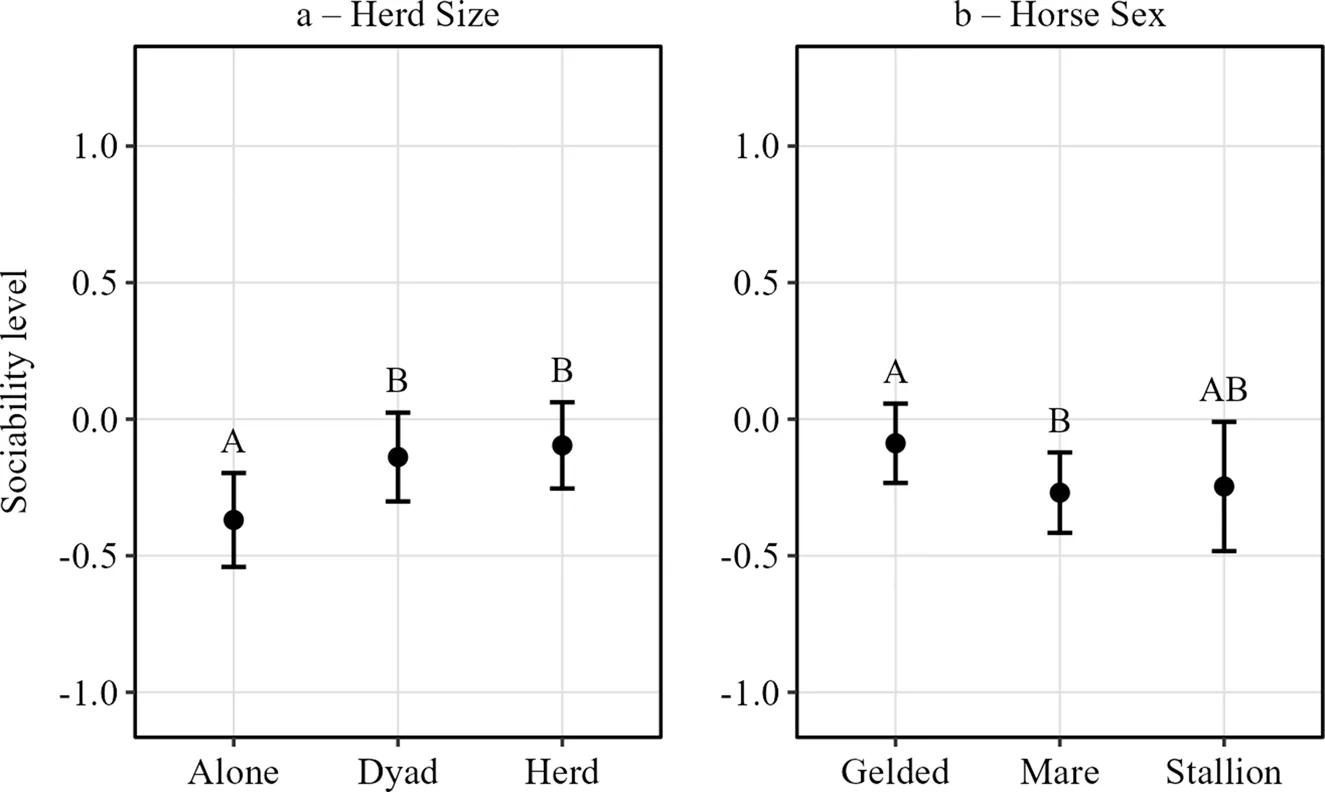
Association of horse sociability score with the (a) herd size and (b) horse sex. Distinct letters signify statistically significant differences among the groups. The means and their associated 95% confidence intervals (error bars) were extracted from the corresponding model using the function emmeans from the ‘emmeans’ package in R.

**Figure 8.**
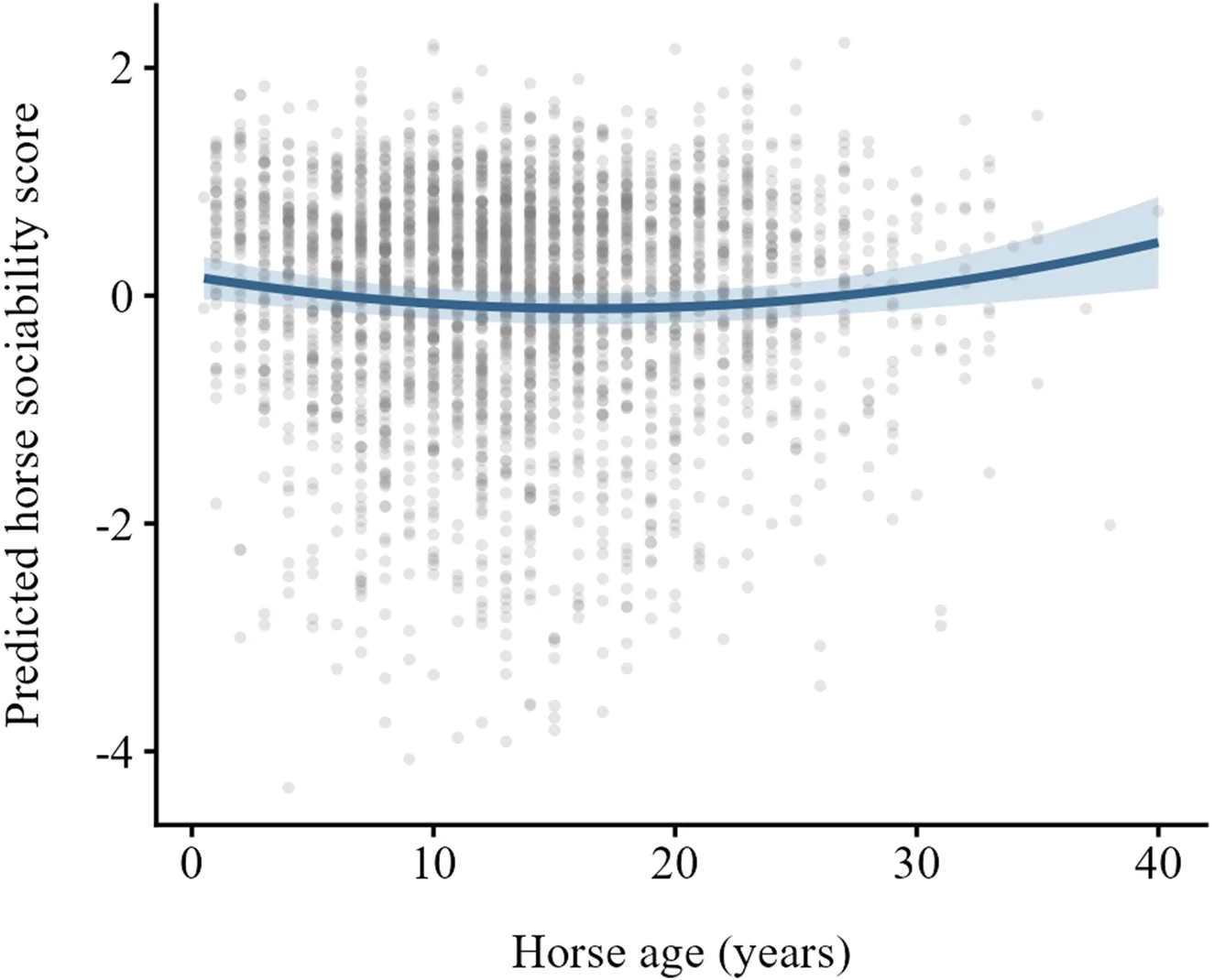
Association of horse sociability score with the horse's age. The blue line demonstrates the predicted horse sociability score throughout the horse's age. The light blue area represents the lower and upper 95% confidence intervals extracted from the corresponding model.

Notable but with minimal association, **the social context of riding** showed a trend towards overall significance (*χ*² = 7.77, *p* = 0.051). Horses ridden by friends or family were significantly more attentive than non-ridden horses (*β* = 0.17, *z* = 2.67, *p* = 0.0076), corresponding to a 2.4% increase across the attentiveness range (figure 4b).

**Ownership history** also contributed significantly to variation in attentiveness (*χ*² = 10.62, *p* = 0.005). Horses purchased directly from a breeder scored approximately 1.9% higher than those with a history of multiple owners (difference = 0.14, *z* = 3.24, *p* = 0.0034) (figure 4c).

#### 3.2.3. Neuroticism

Horse breed and age produced the largest shifts across the neuroticism spectrum, while sex and riding frequency showed smaller but significant associations.

**Horse breed** was strongly associated with neuroticism (*χ*² = 55.73, *p* < 0.001). Warmblood horses exhibited the highest neuroticism scores, corresponding to a medium-to-large positive effect (*β* = 0.45, *z* = 6.20, *p* < 0.001). Warmbloods scored 7.8% higher than draught horses (difference = 0.45, *z* = 6.20, *p* < 0.0001), 4.5% higher than Nordic breeds (difference = 0.26, *z* = 4.50, *p* = 0.0001) and 2.9% higher than horses of unknown breed (difference = 0.17, *z* = 2.92, *p* = 0.029). Ponies also showed elevated neuroticism relative to draught horses, scoring 5.0% higher across the observed range (*β* = 0.29, *z* = 2.96, *p* = 0.003) (figure 5a).

**Horse age** is also significantly associated with neuroticism (*χ*² = 27.35, *p* < 0.001). While the linear age component was not statistically significant (*β* = −1.41, *p* = 0.37), a strong negative quadratic effect was observed (*β* = −5.96, *z* = −4.95, *p* < 0.001), indicating a pronounced nonlinear pattern in which neuroticism varied substantially across the lifespan, with marked changes during middle age (figure 6).

**Horse sex** further contributed to variation in neuroticism (*χ*² = 22.52, *p* < 0.001). Geldings displayed the highest neuroticism scores, scoring 5.6% higher than stallions (difference = 0.33, *z* = 3.27, *p* = 0.003) and 2.6% higher than mares (difference = 0.15, *z* = 4.02, *p* < 0.001). Differences between mares and stallions were smaller and non-significant (difference = 0.17, *z* = 1.76, *p* = 0.18) (figure 5b).

**Riding frequency** was significantly associated with neuroticism (*χ*² = 16.38, *p* = 0.006). This effect was characterized by a small negative linear trend (*β* = −0.11, *z* = −2.14, *p* = 0.033), indicating a lower neuroticism score with higher riding frequency. Post hoc comparisons showed that horses ridden rarely scored 4.0% higher on the neuroticism scale than horses worked intensively four to seven times per week (difference = 0.23, *z* = 3.64, *p* = 0.004), reflecting a small but meaningful difference across the observed range (figure 5c).

#### 3.2.4. Horse sociability

In contrast to human-directed traits, intraspecific sociability was primarily associated with the social environment and sex, while horse age showed a significant nonlinear relationship.

**Herd size** emerged as a strong predictor of sociability towards other horses (*χ*² = 24.22, *p* < 0.001). This relationship was characterized by a small-to-medium positive linear effect (*β* = 0.19, *p* < 0.001), indicating that sociability increased as horses were housed with conspecifics, followed by a small negative quadratic component (*β* = −0.08, *p* = 0.031), suggesting a plateau once horses were no longer housed alone. Post hoc comparisons confirmed that horses kept in a herd scored 4.2% higher on the sociability scale than horses housed alone (difference = 0.27, *z* = −4.91, *p* < 0.0001), while horses housed in dyads scored 3.5% higher than solitary horses (difference = 0.23, *z* = −3.86, *p* = 0.0003). No meaningful difference was observed between horses housed in dyads and those kept in larger herds (differencee = 0.04, *z* = −1.01, *p* = 0.572), indicating that the primary contrast lies between social versus solitary housing rather than group size *per se* (Figure 7a).

**Horse sex** is also significantly associated with sociability towards other horses (*χ*² = 22.58, *p* < 0.001). The effect is small, but geldings exhibited higher levels of intraspecific sociability than mares, with mares scoring 2.8% lower on the sociability scale relative to geldings (*β* = −0.18, *z* = −4.70, *p* < 0.0001). Differences between geldings and stallions were smaller and did not reach statistical significance. No meaningful difference was detected between mares and stallions either (Figure 7b).

**Horse age** showed a significant nonlinear association with sociability towards other horses (*χ*² = 11.56, *p* = 0.003). Although the linear age effect was not significant (*p* = 0.294), a large positive quadratic effect was observed (*β* = 3.34, *p* < 0.001), indicating a U-shaped pattern in which sociability was relatively higher in younger and older horses, with a relative reduction during middle age. This quadratic effect corresponded to approximately 51.1% of the total observed sociability range, highlighting age as an important but non-monotonic contributor to intraspecific social behaviour (Figure 8).

## 4. Discussion

Across the diverse field of equine personality research with substantial variability in questionnaire design and analytical approaches, similar personality factors have been observed to emerge. In their review, Rankins and Wickens [15] identified reactivity, gregariousness, reactivity to humans, sensory sensitivity and locomotor activity as equine personality factors. The four personality factors (human sociability, attentiveness, neuroticism and horse sociability) found in this study align closely with these recurring domains, supporting both its construct validity and its comparability with previous factor-analytic work. The factor score distributions further support the psychometric robustness of the model, with all factors approximately normally distributed. This facilitates linear modelling and meaningful comparisons across individuals and groups. The presence of long tails (up to ±4 s.d.) indicates extreme but behaviourally meaningful trait expressions, as also reported in other equine personality questionnaires [39–41]. Overall, despite differences in terminology across instruments, the present factor structure shows strong convergence with previously identified equine personality domains summarized in systematic and conceptual reviews.

However, when interpreting our results, it is important to keep in mind that 94% of the respondents were female. Consequently, the daily management practices and behavioural ratings reported here primarily reflect a female perspective.

### 4.1. Human sociability

In our analyses, the human sociability factor corresponds to the tendency of a horse to seek, tolerate and enjoy interactions with humans. Horses with low human sociability would avoid or ignore people, especially strangers. They would often be difficult to catch, show little interest in interaction and may appear distrustful or withdrawn. Human contact is tolerated rather than actively sought. In contrast, horses with high human sociability readily approach people and show curiosity, affection and playfulness. They are easy to catch, comfortable with strangers and actively seek human interaction. This factor corresponds closely to traits described as reactivity to humans or gregariousness towards people or sociability/protection towards humans in instruments such as the Equine Personality Test [42] the Horse Personality Questionnaire or the Equine Behavioural Assessment and Research Questionnaire (E-BARQ) [26,39,41]. Reactivity and responsiveness to humans have repeatedly been identified as central trait clusters in questionnaire-based research, and their consistent emergence suggests that horses’ orientation towards human interaction represents a stable and salient personality domain.

Regarding the exploration of the associations between human sociability scores and the relationship, environmental and intrinsic parameters, human sociability was most associated with age, with scores decreasing with horse age and stabilizing after about 20 years of age. This is in line with behavioural observations from Lerch *et al.* [43] where, in a motionless person test, 3–15‑year‑old equids interacted more with the human than those over 15 years old, suggesting a nonlinear age effect with reduced interaction in the oldest individuals. The human sociability factor included positively loaded items of curiosity and playfulness regardless of if it was directed towards humans or not, and young individuals of many species [44,45], including horses [46,47], generally show higher explorative behaviours and playfulness, which could drive this pattern. Additionally, as horses age, the more likely they are to interact with humans in new ways, including riding, training, transport and veterinary procedures, which may not be considered as a positive interaction for the horses [48,49], possibly reducing their interest in humans if the daily interactions do not always guarantee a positive output for them. Ensuring that daily interactions remain rewarding throughout a horse's life is, therefore, important for maintaining positive relationships, especially in older individuals.

Horse sex was also strongly associated with perceived human sociability, with geldings receiving higher ratings than mares. Consistent with this, Lerch *et al.* [43] reported that geldings interacted more frequently with a motionless human. In contrast, other studies have identified little to no behavioural sex differences in human–horse interaction contexts [50–52]. Additionally, the same respondents reported higher avoidant attachment scores towards mares compared with geldings and stallions [29]. These patterns may reflect gendered perceptions of horse temperament rather than true behavioural differences. As most respondents in the present study were women (94.6%), this interpretation is supported by findings from Fenner *et al.* [53], who showed that female riders often hold gendered views of horse temperament. Geldings are commonly perceived as possessing favourable traits, such as calmness, trainability, reliability and predictability, which may be reflected in how geldings and mares are rated in personality surveys. Such perceptions may unintentionally shape welfare outcomes, with geldings potentially receiving more positive handling and opportunities for social interaction than mares.

Owners who spent more frequent quality time with their horses (defined as time spent without making specific demands, where the animal is free to interact or not and often receives positive outcomes like treats and scratches) rated their horses higher in human sociability. While the cross-sectional nature of our data cannot establish a definitive causal direction, these findings point to a strong association that may be bidirectional; owners may naturally dedicate more unstructured quality time to horses they already perceive as gregarious, or conversely, repeated, pleasant interactions initiated by humans (e.g. gentle touch and predictable care) are known to reinforce voluntary approach and relaxed behaviour in animals [54]. This aligns with the systematic review by Hall and Kay [49], who concluded that positive experiences and voluntary approach behaviour to humans are key indicators of good welfare, and that interaction quantity and quality strongly modulate these horse–human dynamics.

Horses with shorter relationships with their owners and those that had been sold multiple times throughout their lives were rated lower in human sociability. This finding aligns with Liehrmann *et al.* [51], who reported that shorter relationship duration and frequent owner changes influenced horses’ responses during novel object tests conducted with human handlers. These results suggest that reduced familiarity with humans and repeated changes in environment may negatively affect horses’ confidence and alter their human-directed responses. Our findings extend this work by indicating that such life-history factors may also be reflected in horses’ general behavioural tendencies towards humans.

Finally, horses with higher sociability scores were more likely to be ridden by friends and family members of the owner. This may indicate that more sociable horses are perceived as more trustworthy, leading owners to feel more comfortable allowing people they care about to ride them.

### 4.2. Attentiveness

The **attentiveness** factor represents the horse's capacity to focus on, respond to and cooperate with human cues and handling. Horses with low attentiveness scores are easily distracted, inconsistent and uncooperative during handling or training. They may become irritable or frustrated under constraint and respond poorly to human cues. Highly attentive horses are focused, responsive and consistent. They follow cues reliably, remain calm when handled and are easy to work with during training. This factor aligns with traits often described as reliability and obedience under human control. Comparable constructs appear across questionnaires under labels such as reliability, obedience, understanding or trainability [15,41,55].

Attentiveness scores were predominantly positively associated with horse age. Age may also correlate with the level of training received, as younger horses that are still in training may be more likely to be rated highly on items such as distractibility or frustration. In contrast, more experienced horses may show higher levels of cooperative behaviour and be less reactive to external stimuli owing to habituation. Liehrmann *et al.* [52] found that in an object-choice task involving human referential communication, task success increased with age, with older horses (over 19 years old) achieving the highest success rates. Similarly, Proops *et al.* [56] reported that while young horses could successfully use pointing and touching cues to locate a rewarded bucket, they failed to respond to more subtle cues such as body orientation, gaze, elbow pointing or tapping. If horses’ ability to interpret nuanced human body signals improves with age and experience, older horses may, therefore, be more likely to receive higher ratings on behavioural items related to cooperation and attentiveness.

Attentiveness was also positively associated with riding frequency, with horses ridden four to seven times per week receiving higher attentiveness scores. As with age, riding frequency may reflect differences in training exposure, such that more frequently ridden horses benefit from greater training consistency and are, therefore, more likely to score highly on items related to cooperative work behaviours. Conversely, it is also possible that horses rated as highly attentive are preferentially selected or retained by regular riders, meaning that horses with this temperament are more likely to be ridden frequently. Supporting this, in Visser *et al.* [54], riders preferred to ride horses they themselves had assessed as ‘attentive to the rider's aids’. Similarly, in a questionnaire of 197 riders on ‘ideal horse’ characteristics, behaviour during riding and handling was the most important category which included similar items, such as manageability, ease of handling and sociability, all closely tied to attentive, compliant behaviour [57]. Large-scale E‑BARQ data further support this, showing that rider and management characteristics, such as the number and type of regular riders, are systematically related to a horse's compliance and responsiveness to aids [58,59]. These data also indicate that traits like being ‘safe’ and ‘easy to ride’ cluster alongside other compliant behaviours that make horses attractive mounts for regular use, reinforcing the idea that rider preferences and selection pressures shape which horses are preferentially retained and used most intensively by frequent riders [60].

### 4.3. Neuroticism

The **neuroticism** factor is linked to the degree of horse emotional reactivity, anxiety and sensitivity to stress. Horses scoring low on neuroticism are described as calm, confident and emotionally stable. They show little fear or anxiety and cope well with novel or challenging situations. Highly neurotic horses are anxious, fearful and easily stressed. They show strong emotional reactions and increased reactivity to environmental changes. This factor directly parallels dimensions, such as nervousness, anxiousness, excitability, emotionality or fearfulness, which are among the most robust and consistently reported traits in equine personality research [15] and are widely documented across species [61] (cats: [62]; ferrets: [63]; dogs: [64]). The E-BARQ captures equine boldness, defined as the horse's reaction to the unfamiliar or unexpected, as a core dimension, which constitutes roughly the inverse of our neuroticism factor [41].

Not surprisingly, breed appeared as the strongest association with neuroticism. Similarly, in the review of [15], the authors also report that reactivity/sensory sensitivity and anxiousness are among the traits that are most clearly influenced by breed and heritable factors. Our results show that draught horses score the lowest in neuroticism, followed by Nordic horses, ponies and then warmbloods with the highest scores. Our results somewhat align with those of Burattini *et al.* [65] who observed that Thoroughbreds were less bold than cross-breed horses and that draught horses and ponies were bolder. Sackman and Houpt [66] also found clear breed differences, with Arabians, Thoroughbreds and Saddlebreds (highly represented in our warmblood category) rated as the most nervous and draughts as the least nervous in a survey. Modern warmbloods are intensively selected for athletic sport performance and responsive mental attitude [67,68]. Within warmblood sport populations, higher proportions of Thoroughbred ancestry are associated with higher baseline heart rate and greater fear reactivity to novel stimuli, and warmblood and Thoroughbred sport horses show higher hypothalamic–pituitary–adrenal axis reactivity than cold‑blooded breeds [69,70].

Interestingly, although the effect size was small, both younger and older horses scored lower in neuroticism than ‘middle-aged’ horses, with a peak observed at approximately 15 years of age. This pattern is partially consistent with evidence that younger horses tend to show more exploratory behaviour [46,47] and may, therefore, be perceived by owners as bolder, leading to lower ratings on neuroticism-related items. However, Baragli *et al.* [71] reported that although younger horses displayed more exploratory behaviour towards a novel object in a suddenness test, they also exhibited stronger avoidance responses than older individuals. It is important to note that many studies examining age effects on horse behaviour rely on strictly linear models and do not consider potential quadratic relationships, such as the pattern observed in the present study, which may limit direct comparability of findings. Very few studies include sufficient geriatric horses (over 18 years old) in their samples to draw strong conclusions. In our study, horses may be rated as less fearful once retired and consequently exposed to fewer potentially aversive or challenging stimuli than actively working horses. As a result, owners of older horses may observe fewer fear-related behaviours, which could contribute to lower neuroticism ratings compared with those assigned to ridden, working horses that encounter a wider range of external stimuli. Older horses commonly develop ocular lesions, such as cataracts and senile retinopathy, and the prevalence of these changes increases with age [72–74]. Even when owners still perceive their horses as visual, this age‑related vision decline could reasonably make some older horses less reactive to external visual stimuli like distant movement or subtle environmental changes. Another explanation comes from the E-BARQ data, analysed in Burattini *et al.* [65], which report that older horses are rated by owners as bolder and interpret this as a consequence of accumulated experience, habituation and greater training exposure.

Riding frequency and sex were also associated with neuroticism scoring, but with smaller magnitude. Horses ridden more frequently scored lower than those rarely ridden. It is in line with the review conclusions of [15], which emphasizes that training intensity, exposure and handling practices can modify the expression of personality traits such as reactivity and fearfulness, even when underlying temperament is genetically influenced. This is consistent with E‑BARQ data showing that, with increasing age and accumulated training, horses become bolder and more responsive to rider cues, presumably through habituation and repeated exposure to varied stimuli [58,65]. It is also possible that fearful horses are not preferred to be ridden regularly, which could influence our results. There may be a strong implication of horse personality regarding training management and horse selection depending on the tasks they are used for. As for the sex difference, we found a small difference with gelding scoring a bit higher than mares or stallions in neuroticism. Sex effects related to fearful behaviour is contradictory, as some studies using fear and novel object tests align with our findings [22,75], while others observed no sex differences [51,76] or stallions to be more reactive than mares [77]. While these conflicting patterns make a definitive interpretation challenging, our data point to a potential behavioural mechanism. In our sample, geldings also scored the highest in human sociability. If owners perceive their geldings as more social and agreeable, it may foster a higher level of trust, leading to increased likelihood of exposing these horses to novel or potentially fearful stimuli in varied environments as in Fenner *et al.* [53], who reported that female riders often view geldings as more suitable for show jumping, dressage and trail riding than mares.

### 4.4. Horse sociability

The **horse sociability** factor represents the horse's tendency to engage in affiliative, non-aggressive inter actions with other horses. Horses with low horse sociability are more likely to be aggressive, invasive or controlling towards other horses. They may struggle with group integration and show frequent social conflict. Highly horse-sociable horses are described as showing more affiliative and submissive behaviour and being well-integrated within the group. They show low aggression and maintain positive relationships with conspecifics. This factor relates to what is also called gregariousness, affiliative behaviour towards conspecifics or non-human social confidence in the literature [41,42] but not always analytically separable from human-directed sociability [26,28,39]. This limited separability in earlier studies may be partly attributable to smaller questionnaires, typically comprising 25–30 items, which reduce factor overdetermination and increase the likelihood that distinct sociability domains merge into broader factors. In contrast, the clear distinction between human sociability and horse sociability observed in the present study is probably facilitated by the large sample size (*n* > 2000) and extensive item set (*n* = 52). Larger samples (*n* > 300) improve the stability and precision of factor loadings, enhancing the ability to differentiate correlated latent traits [78], while a higher number of items allows more specific constructs to emerge.

Not surprisingly, horse sociability scores were mainly driven by their social life: horses housed mainly alone scored lower on sociability than horses housed with conspecifics. This is in line with E‑BARQ data, where lower non‑human social confidence scores are interpreted as an indication that horses may benefit from social enrichment with conspecifics [41]. The most common reasons for housing horses singly are linked to perceived risks and practical constraints rather than doubts about the benefits of social, outdoor living. Owners primarily cite concerns about injuries, group feeding and the management of high-value, stallion or competition horses, leading them to choose boxes or individual paddocks despite generally agreeing that group turnout and outdoor access are better for welfare [79–81]. It is possible that horses housed alone are rated lower because horse owners do not witness affiliative behaviour, although respondents had the option to precise ‘I don't know’. One reason horses housed alone may score lower in horse sociability is because deprivation from conspecific may then alter their social skill, as shown in stallions by Christensen *et al.* [82], where prolonged individual housing increased the risk and intensity of agonistic behaviours when horses are put back into contact. On the other hand, we cannot exclude that owners may have witnessed strong agonistic interactions from their horses and then decided to house them individually to prevent injuries. Taken together, these perspectives highlight important welfare implications regardless of the direction of the relationship between personality and social housing. If housing decisions are influenced by perceived personality traits, some horses may be systematically excluded from social environments, leading to unequal welfare outcomes despite social contact being a fundamental need. Conversely, if the social environment shapes personality, restrictive housing may contribute to the development of less adaptive behavioural profiles, such as reduced sociability or heightened agonistic responses, which can, in turn, justify continued isolation. In both cases, a feedback loop may emerge in which management practices and personality reinforce one another, underscoring the importance of considering the long-term welfare consequences of social housing decisions and individual needs of horses.

Finally, age and sex were also associated with horse sociability, with younger and older horses scoring the highest and mares scoring lower than geldings. Age associations are somewhat supported by the observations of van Dierendonck *et al.* [83] in Icelandic horse herds where dominance rank increased with age and older mares formed long‑lasting affiliative bonds and subgroups, while very young horses show high levels of allogrooming and play. Regarding the sex differences, a review of equine social behaviour [84] notes that both sexes engage in affiliative interactions, and that domestic group compositions (mare–gelding, bachelor groups and mixed herds) can all support stable bonds if management is adequate.

### 4.5. Other parameters

Interestingly, groundwork frequency (training from the ground with a lead rope or at liberty without riding the horse) was never selected in the models, and the use of bitless headgear had no meaningful association with any of the personality factors. Groundwork, including in-hand and lunging work, has long existed in European traditions, but modern ‘natural horsemanship’ approaches, particularly liberty and round‑pen work, have grown in popularity recently [53,85,86]. Bitless bridles, though not new (e.g. hackamores and cavessons), remain rare overall, especially in rule-bound sports like dressage, but are more common in American Western and alternative disciplines [87,88]. Surveys and ethological studies show that both bitless tack and liberty/round‑pen methods are closely associated with natural horsemanship, equine-assisted activities, welfare- or science-branded training and relationship-centred approaches, while remaining uncommon in traditional English competition contexts [53,87,89,90]. Liehrmann *et al.* [27] observed that, in the same cohort of respondents, higher frequency of groundwork training and the use of bitless gear were associated with owner's lower avoidant attachment style and higher scores in openness personality trait, showing that these parameters depend on the horse owner's interest for less traditional practices [27]. This interpretation is consistent with the development of E‑BARQ, where numerous management and training items were removed because they attracted low response rates or showed very limited variance, indicating that some practices are confined to specific subcultures rather than broadly shaping behaviour across the population [41]. In this context, our finding that groundwork frequency and bitless headgear use were unrelated to personality factors is compatible with the idea that these variables mainly capture owner training philosophy rather than stable horse traits.

Time spent at pasture was not retained in any of the models, indicating no significant association with either personality factor. In our survey, pasture was deliberately defined in a simple way—as a grazing field larger than one hectare—following Liehrmann *et al.* [52], who reported behavioural associations using a similar definition. In their study, however, pasture time was highly correlated with herd size, preventing disentanglement of these effects. This confounding was not present in our data, making the absence of an association in our results unexpected, given the well-established link between pasture access and horse welfare. Numerous studies have shown that increased time at pasture, providing space for movement and continuous access to forage, reduces stereotypic and aggressive behaviours and promotes natural locomotion and social interactions, thereby better meeting horses’ species-typical ecological needs [5,7,91]. Seasonal alternation between pasture and indoor housing has also been shown to influence behaviour: summer pasture periods markedly reduce stereotypies and aggression, while the return to individual stabling triggers short-term increases in withdrawal, hypervigilance and stereotypic behaviours, with most welfare benefits disappearing within weeks [13,92]. Based on this literature, an association between pasture time and traits, such as sociality or neuroticism, could have been expected. However, a limitation of our approach is that the broad ordinal categories used to measure pasture time may have lacked the resolution to detect these effects across a highly disparate, geographically diverse sample. Macroclimatic differences mean that pasture utility varies drastically by region; for example, the harsh, dark winters of Fennoscandia and Canada restrict outdoor grazing to entirely different seasonal dynamics and forage qualities compared with the arid or more temperate conditions. Future studies should, therefore, consider more detailed assessments of pasture conditions, including size, terrain variability, availability of forage beyond grass (e.g. hedges or hay) and access to shelter, to better capture the complexity of pasture environments and climate and their potential association with horse consistent behaviour.

### 4.6. Limitations

Demographic factors, such as ethnicity, social class, gender and family status, shape people's experiences with and perspectives towards animals and, thus, influence human–animal relationships [93]. Although the survey was distributed internationally, limiting participation to English, French and Finnish speakers probably biased the sample towards respondents from Western, educated, industrialized, rich, and democratic societies—a well-recognized limitation in psychological research [94,95]. Resource constraints prevented translation into additional languages, underscoring the need for future studies to include a wider linguistic range to achieve greater global representativeness. Moreover, voluntary participation may have introduced additional self-selection biases.

A further limitation is the pronounced gender imbalance in the sample, with 94.6% of respondents identifying as female, reflecting patterns commonly observed in equestrian research [53,88]. Our results may then suffer from a gender bias, as Anzulewicz *et al.* [59] found that the biological sex of handlers and riders was associated with differences in horses’ reported social confidence, compliance and touch sensitivity, with horses ridden by male humans more likely to be described as difficult to catch or defensive. In addition, the study design did not allow for multiple raters per horse or repeated assessments over time. As a result, it may have introduced subjective biases, and the findings also only reflect owners’ perceptions at a single time point rather than stable or long-term behavioural profiles. Finally, the cross-sectional nature of the study precludes causal inference. While associations between personality ratings and environmental or relationship factors can be identified, the directionality of these relationships remains unclear. Longitudinal and multi-rater studies are, therefore, needed to clarify causal pathways and to better capture the dynamic interactions between owner characteristics, management practices and equine personality over time.

## 5. Conclusion

The four personality factors (human sociability, attentiveness, neuroticism and horse sociability) identified in our study align with the overall literature on horse personality. Expectedly, they were primarily associated with intrinsic factors, such as age, breed or sex. However, our study also demonstrates that management practices and relationship parameters—such as housing style, frequency and quality of human interaction, duration of ownership and riding exposure—can have substantial associations with personality traits. These findings are consistent with the view that personality is not solely fixed by intrinsic traits but may also be expressed differently depending on environmental conditions, social opportunities and human engagement. Interindividual variation in the personality of horses may also influence management, interactions and training decisions. Understanding these interactions is crucial because they have direct implications for welfare. Future research should aim to disentangle the relative contributions of personality and environmental or relational factors, to inform management practices that optimize both behavioural development and overall well-being.

## Supplementary Material



## Data accessibility

The data as well as the horse personality survey in English, Finnish and French and the R script are freely available in an online repository [96].

## Declaration of AI use

Programs such as Grammarly and ChatGPT were used solely for language editing purposes, including grammar checking, spelling correction and improving sentence clarity and readability. These tools were not used to generate scientific content, ideas, analyses or interpretations. For example, as the authors are non-native English speakers, ChatGPT was occasionally used to suggest alternative phrasings when sentences were perceived as overly complex or heavy, but preserving the original meaning. All revisions were reviewed and approved by the authors, who remain fully responsible for the final wording and content of the manuscript. The use of other AI-assisted tools in this manuscript was limited to supporting the literature review and contextualization of results. AI tools used were Consensus (pro version) and ResearchRabbit (online free version of the platform available at the time (2023–2025)). Consensus was used as a complementary literature discovery to ensure that relevant studies were not overlooked. Specifically, it was used to check whether findings discussed in the manuscript were reported as similar or contradictory in the existing literature and identify whether comparable results existed for the same species or for other mammalian taxa. ResearchRabbit was used solely for reference management and literature discovery, particularly to organize papers by topic and identify potentially relevant studies through the ‘similar works’ network visualization feature. This AI tool, designed for research, does not require specific prompts and does not provide printable outputs. These tools were used in conjunction with standard manual literature searches, including structured string-based searches in Google Scholar and Scopus, which remained the primary methods for identifying and selecting references. Typical prompts used in Consensus App included requests such as whether similar or contradictory findings had been reported in the literature for a given result, species or broader taxonomic group. AI-generated outputs were used only as informational guidance. All suggested papers, claims and summaries were independently verified by accessing and reading the original publications. No AI content was included without human review and verification. AI tools were used intermittently during the literature review and discussion phases of the project between 2023 and the finalization of the manuscript. Overall, AI tools functioned strictly as supportive discovery and organizational aids, with all scholarly judgement, interpretation and final manuscript content determined by the authors. No AI has been used to create, refine or edit displays and visualizations (such as figures) in our paper.
