# Supplementary material for: Management practices and human–horse relationships are associated with horse personality traits
Source: R Soc Open Sci. 2026 Sep 9;13(9):260116. doi: 10.1098/rsos.260116 (PMC13561068; doi:10.1098/rsos.260116)
Supplement: Supplementary file 1 [file rsos.260116.zip › rsos.260116/rsos260116_si_001.docx]

**Electronic Supplementary Material**

Table S1: Type II Wald χ² Analysis of Variance for Gaussian Generalized Linear Mixed Models Predicting Personality Factors

| model | term | statistic | df | p.value |
| --- | --- | --- | --- | --- |
| human sociability | Quality time freq | 23.32 | 3 | <0.001 |
|  | Headgear | 11.97 | 3 | 0.007 |
|  | Riders context | 12.90 | 3 | 0.005 |
|  | Previous owners | 9.511 | 2 | 0.009 |
|  | Relationship length | 22.90 | 3 | <0.001 |
|  | Horse sex | 78.24 | 2 | <0.001 |
|  | poly(Horse age, 2) | 189.00 | 2 | <0.001 |
| neuroticism | Riding freq | 16.38 | 5 | 0.006 |
|  | Previous owners | 15.57 | 2 | <0.001 |
|  | Relationship length | 7.034 | 3 | 0.071 |
|  | Horse sex | 22.52 | 2 | <0.001 |
|  | Breed cat | 55.73 | 4 | <0.001 |
|  | poly(Horse age, 2) | 27.35 | 2 | <0.001 |
| attentiveness | Riding freq | 28.82 | 5 | <0.001 |
|  | Quality time freq | 10.55 | 3 | 0.014 |
|  | Riders context | 7.76 | 3 | 0.051 |
|  | previous owners | 10.61 | 2 | 0.005 |
|  | poly(Horse age, 2) | 128.63 | 2 | <0.001 |
| horse sociability | Previous owners | 4.04 | 2 | 0.132 |
|  | Herd size | 24.21 | 2 | <0.001 |
|  | Horse sex | 22.57 | 2 | <0.001 |
|  | poly(Horse age, 2) | 11.55 | 2 | 0.003 |

Table S2: Fixed-Effect Parameter Estimates and 95% Confidence Intervals for Personality Factor Models

| 1. **model** |  | term | estimate | std.error | Ratio | p.value |
| --- | --- | --- | --- | --- | --- | --- |
| Human Sociability | (Intercept) | | -0.110 | 0.062 | -1.767 | 0.077 |
|  | Quality Time Freq | quality_time_freq.L | 0.225 | 0.062 | 3.634 | <0.001 |
|  |  | quality_time_freq.Q | -0.061 | 0.058 | -1.058 | 0.290 |
|  |  | quality_time_freq.C | 0.108 | 0.054 | 1.989 | 0.047 |
|  | Headgear | Bitless and bit | 0.021 | 0.043 | 0.484 | 0.628 |
|  |  | Bitless only | 0.132 | 0.109 | 1.208 | 0.227 |
|  |  | Not ridden | -0.152 | 0.053 | -2.852 | 0.004 |
|  | Riders Context | Friends/familly | 0.202 | 0.060 | 3.381 | 0.001 |
|  |  | Shared with others | 0.170 | 0.069 | 2.456 | 0.014 |
|  |  | Exclusive rider | 0.120 | 0.048 | 2.511 | 0.012 |
|  | Previous Owners | Bought from breeder | 0.131 | 0.045 | 2.944 | 0.003 |
|  |  | Owner is breeder | 0.150 | 0.069 | 2.169 | 0.030 |
|  | Relationship Length | relationship_length_cat.L | 0.285 | 0.062 | 4.560 | <0.001 |
|  |  | relationship_length_cat.Q | 0.037 | 0.041 | 0.880 | 0.379 |
|  |  | relationship_length_cat.C | 0.028 | 0.034 | 0.828 | 0.408 |
|  | Horse sex | Mare | -0.323 | 0.037 | -8.803 | <0.001 |
|  |  | Stallion | -0.251 | 0.097 | -2.593 | 0.010 |
|  | Horse Age | poly 1 | -20.451 | 1.514 | -13.509 | <0.001 |
|  |  | poly 2 | 5.793 | 1.093 | 5.299 | <0.001 |
| Neuroticism | (Intercept) | | -0.210 | 0.098 | -2.139 | 0.032 |
|  | Riding frequency | riding_freq.L | -0.112 | 0.053 | -2.137 | 0.033 |
|  |  | riding_freq.Q | -0.093 | 0.051 | -1.824 | 0.068 |
|  |  | riding_freq.C | -0.079 | 0.053 | -1.494 | 0.135 |
|  |  | riding_freq^4 | 0.062 | 0.053 | 1.154 | 0.249 |
|  |  | riding_freq^5 | -0.060 | 0.054 | -1.122 | 0.262 |
|  | Previous Owners | Bought from breeder | -0.179 | 0.046 | -3.930 | <0.001 |
|  |  | Owner is breeder | -0.105 | 0.071 | -1.477 | 0.140 |
|  | Relationship Length | relationship_length_cat.L | -0.136 | 0.064 | -2.122 | 0.034 |
|  |  | relationship_length_cat.Q | 0.041 | 0.043 | 0.964 | 0.335 |
|  |  | relationship_length_cat.C | 0.037 | 0.035 | 1.061 | 0.289 |
|  | Horse Sex | Mare | -0.151 | 0.038 | -4.016 | <0.001 |
|  |  | Stallion | -0.325 | 0.099 | -3.271 | 0.001 |
|  | Breed | Nordic | 0.190 | 0.087 | 2.184 | 0.029 |
|  |  | pony | 0.289 | 0.098 | 2.958 | 0.003 |
|  |  | Warm-blooded | 0.450 | 0.073 | 6.195 | <0.001 |
|  |  | Unknown | 0.280 | 0.086 | 3.244 | 0.001 |
|  | Horse Age | poly 1 | -1.406 | 1.554 | -0.905 | 0.366 |
|  |  | poly 2 | -5.963 | 1.205 | -4.948 | <0.001 |
| Attentiveness | (Intercept) | | -0.190 | 0.054 | -3.542 | <0.001 |
|  | Riding frequency | riding_freq.L | 0.213 | 0.053 | 3.990 | <0.001 |
|  |  | riding_freq.Q | 0.098 | 0.052 | 1.880 | 0.060 |
|  |  | riding_freq.C | 0.001 | 0.054 | 0.017 | 0.986 |
|  |  | riding_freq^4 | 0.002 | 0.055 | 0.038 | 0.969 |
|  |  | riding_freq^5 | -0.136 | 0.055 | -2.464 | 0.014 |
|  | Quality Time frequency | quality_time_freq.L | 0.010 | 0.065 | 0.159 | 0.873 |
|  |  | quality_time_freq.Q | 0.137 | 0.061 | 2.248 | 0.025 |
|  |  | quality_time_freq.C | -0.024 | 0.057 | -0.421 | 0.674 |
|  | Riders Context | Friends/familly | 0.166 | 0.062 | 2.671 | 0.008 |
|  |  | Shared with others | 0.072 | 0.072 | 0.997 | 0.319 |
|  |  | Exclusive rider | 0.047 | 0.050 | 0.937 | 0.349 |
|  | Previous Owners | Bought from breeder | 0.137 | 0.042 | 3.242 | 0.001 |
|  |  | Owner is breeder | 0.088 | 0.064 | 1.376 | 0.169 |
|  | Horse age | poly 1 | 11.741 | 1.062 | 11.051 | <0.001 |
|  |  | poly 2 | -2.756 | 1.185 | -2.327 | 0.020 |
| Horse Sociability | (Intercept) | | 0.015 | 0.072 | 0.208 | 0.835 |
|  | Previous Owners | bought from breeder | -0.079 | 0.043 | -1.862 | 0.063 |
|  |  | Owner is breeder | -0.086 | 0.064 | -1.333 | 0.183 |
|  | Herd Size | herd_size.L | 0.193 | 0.039 | 4.906 | <0.001 |
|  |  | herd_size.Q | -0.077 | 0.036 | -2.151 | 0.031 |
|  | Horse Sex | Mare | -0.181 | 0.038 | -4.700 | <0.001 |
|  |  | Stallion | -0.158 | 0.102 | -1.548 | 0.122 |
|  | Horse Age | poly 1 | -1.098 | 1.046 | -1.049 | 0.294 |
|  |  | poly 2 | 3.334 | 1.010 | 3.301 | 0.001 |

Table S3: Standardized Parameter Estimates (β) and 95% Confidence Intervals for Personality Factor Models

| model | Predictor | Term | Std Coefficient | 95% CI_low | 95% CI_high |
| --- | --- | --- | --- | --- | --- |
| Human Sociability | (Intercept) | | -0.110 | -0.230 | 0.011 |
|  | Quality Time Freq | quality_time_freq.L | 0.222 | 0.102 | 0.342 |
|  |  | quality_time_freq.Q | -0.061 | -0.173 | 0.052 |
|  |  | quality_time_freq.C | 0.106 | 0.002 | 0.211 |
|  | Headgear | Bitless and bit | 0.020 | -0.062 | 0.103 |
|  |  | Bitless only | 0.131 | -0.081 | 0.343 |
|  |  | Not ridden | -0.150 | -0.253 | -0.047 |
|  | Riders Context | Friends/familly | 0.200 | 0.084 | 0.316 |
|  |  | Shared with others | 0.168 | 0.034 | 0.302 |
|  |  | Exclusive rider | 0.119 | 0.026 | 0.212 |
|  | Previous Owners | Bought from breeder | 0.130 | 0.043 | 0.216 |
|  |  | Owner is breeder | 0.148 | 0.014 | 0.283 |
|  | Relationship Length | relationship_length_cat.L | 0.282 | 0.161 | 0.403 |
|  |  | relationship_length_cat.Q | 0.036 | -0.044 | 0.117 |
|  |  | relationship_length_cat.C | 0.028 | -0.038 | 0.093 |
|  | Horse Sex | Mare | -0.320 | -0.391 | -0.248 |
|  |  | Stallion | -0.248 | -0.436 | -0.061 |
|  | Horse Age | poly 1 | -20.230 | -23.165 | -17.295 |
|  |  | poly 2 | 5.730 | 3.611 | 7.849 |
| Neuroticism | (Intercept) | | -0.210 | -0.404 | -0.016 |
|  | Riding Freq | riding_freq.L | -0.113 | -0.217 | -0.009 |
|  |  | riding_freq.Q | -0.094 | -0.195 | 0.007 |
|  |  | riding_freq.C | -0.079 | -0.183 | 0.025 |
|  |  | riding_freq^4 | 0.062 | -0.043 | 0.168 |
|  |  | riding_freq^5 | -0.061 | -0.167 | 0.045 |
|  | Previous Owners | Bought from breeder | -0.181 | -0.271 | -0.091 |
|  |  | Owner is breeder | -0.106 | -0.246 | 0.035 |
|  | Relationship Length | relationship_length_cat.L | -0.137 | -0.264 | -0.010 |
|  |  | relationship_length_cat.Q | 0.041 | -0.043 | 0.126 |
|  |  | relationship_length_cat.C | 0.037 | -0.031 | 0.105 |
|  | Horse Sex | Mare | -0.152 | -0.226 | -0.078 |
|  |  | Stallion | -0.327 | -0.524 | -0.131 |
|  | Breed | breed_catNordic | 0.192 | 0.020 | 0.364 |
|  |  | breed_catpony | 0.291 | 0.098 | 0.483 |
|  |  | breed_catWarm-blooded | 0.453 | 0.310 | 0.596 |
|  |  | breed_catUnknown | 0.282 | 0.112 | 0.453 |
|  | Horse Age | poly 1 | -1.417 | -4.485 | 1.651 |
|  |  | poly 2 | -6.005 | -8.384 | -3.626 |
| Attentiveness | (Intercept) | | -0.188 | -0.293 | -0.083 |
|  | Riding Freq | riding_freq.L | 0.211 | 0.108 | 0.315 |
|  |  | riding_freq.Q | 0.098 | -0.004 | 0.199 |
|  |  | riding_freq.C | 0.001 | -0.104 | 0.105 |
|  |  | riding_freq^4 | 0.002 | -0.104 | 0.108 |
|  |  | riding_freq^5 | -0.135 | -0.242 | -0.028 |
|  | Quality Time Freq | quality_time_freq.L | 0.010 | -0.116 | 0.136 |
|  |  | quality_time_freq.Q | 0.136 | 0.017 | 0.255 |
|  |  | quality_time_freq.C | -0.024 | -0.134 | 0.087 |
|  | Riders Context | Friends/familly | 0.165 | 0.044 | 0.286 |
|  |  | Shared with others | 0.072 | -0.069 | 0.213 |
|  |  | Exclusive rider | 0.047 | -0.051 | 0.144 |
|  | Previous Owners | Bought from breeder | 0.136 | 0.054 | 0.219 |
|  |  | Owner is breeder | 0.088 | -0.037 | 0.212 |
|  | Horse Age | poly 1 | 11.677 | 9.606 | 13.748 |
|  |  | poly 2 | -2.741 | -5.051 | -0.432 |
| Horse Sociability | (Intercept) | | 0.016 | -0.126 | 0.158 |
|  | Previous Owners | Bought from breeder | -0.080 | -0.163 | 0.004 |
|  |  | Owner is breeder | -0.086 | -0.213 | 0.041 |
|  | Herd Size | herd_size.L | 0.194 | 0.116 | 0.271 |
|  |  | herd_size.Q | -0.077 | -0.147 | -0.007 |
|  | Horse Sex | Mare | -0.181 | -0.257 | -0.106 |
|  |  | Stallion | -0.159 | -0.359 | 0.042 |
|  | Horse Age | poly 1 | -1.101 | -3.158 | 0.956 |
|  |  | poly 2 | 3.344 | 1.358 | 5.329 |
