# Supplementary material for: Management practices and human–horse relationships are associated with horse personality traits
Source: R Soc Open Sci. 2026 Sep 9;13(9):260116. doi: 10.1098/rsos.260116 (PMC13561068; doi:10.1098/rsos.260116)
Supplement: Supplementary file 1 [file rsos.260116.zip › rsos.260116/rsos260116_si_002.docx]

Prompts and outputs regarding the use of Research Rabbit and consensus app, two AI tools designed for helping researchers with their literature review.

**Research rabbit**

Link: <https://app.researchrabbit.ai/>

Research Rabbit was used in 2023 to identify the literature relevant to the development of this study. The platform does not require the use of prompts and does not provide printable outputs.

Figure 1 illustrates an example of the literature maps generated by Research Rabbit when searching for papers similar to Rankins and Wickens (2020). Papers shown in red represent those already included in the collection created for the horse personality survey and indicate their degree of similarity to the source paper. Papers shown in white represent additional relevant studies that were not previously included in the collection. By selecting individual nodes, basic information such as the title, publication year, abstract, and DOI can be accessed, enabling further review of the identified papers.

reference of the paper used as a source: Rankins EM, Wickens CL. A systematic review of equine personality. *Appl Anim Behav Sci*. 2020;231:105076. doi:10.1016/j.applanim.2020.105076

Figure 1: Map generated by Reseach Rabbit for the search for similar work as the study of Rankins and Wickens (2020). Map generated on the 29.01.2026 for the ESM.

**Prompts used with consensus app (pro version):**

Consensus was used during the year 2025 to implement the literature in the discussion of the manuscript.

Consensus was used as a complementary literature discovery to ensure that relevant studies were not overlooked. Specifically, it was used to check whether findings discussed in the manuscript were reported as similar or contradictory in the existing literature; Identify whether comparable results existed for the same species or for other mammalian taxa. AI-generated outputs were used only as informational guidance. All suggested papers, claims, and summaries were independently verified by accessing and reading the original publications. No AI content was included without human review and verification.

Link: https://consensus.app/

Prompt 1 : 08.11.2025

Is there any study investigating the amount of unwanted behaviour while ridden in mare in association with their ovulatory and hormonal cycle?

Output 1:

**Mare behaviour, riding, and the oestrous/ovulatory cycle: what research shows**

Overall, **there is very little direct, controlled research linking *ridden* unwanted behaviour in mares to specific phases of the ovulatory or hormonal cycle**. Most work either describes sexual/oestrus behaviour generally or examines ovariectomy and ovarian tumours in mares presented for “behaviour problems”.

**What is known about behaviour across the cycle**

Several studies document changes in behaviour and physiology across the oestrous cycle, but not under saddle:

- Detailed work shows systematic changes in cardiac activity, interactions with humans/conspecifics, and locomotor activity between oestrus and dioestrus, but these were measured at liberty, not when ridden (Stachurska et al., 2023).
- Classic ethological and endocrine studies describe estrus vs diestrus sexual behaviour (approach, urination, tail raising, “winking”) and note that estrous intensity peaks near ovulation, but again not under saddle (Crowell‐Davis, 2007; Asa et al., 1979; Asa & Asa, 1986).
- A small study found **oestrous vs dioestrous stage did not change responses** in a novel object and isolation test in “problem” vs control mares (Hedberg et al., 2005).

**Hormones, ovaries, and “behaviour mares”**

Where riding behaviour is mentioned, it is usually in mares presented for suspected “ovary/oestrus-related” problems:

- Retrospective ovariectomy series report owner‑rated improvement in ground behaviour and **rideability** after bilateral ovariectomy, both in mares with normal ovaries and those with granulosa(-theca) cell tumours (Melgaard et al., 2020; Straticò et al., 2023). These are uncontrolled, subjective owner reports and not tied to specific cycle stages.
- A large retrospective hormone‑profile study (n=2914) showed that, apart from stallion‑like behaviour, **most abnormal or aggressive behaviours were *not* associated with elevated ovarian hormones** typical of granulosa cell tumours (Huggins et al., 2023).
- A review of oestrus‑suppression in mares stresses that many “oestrus‑related” behaviour complaints are actually due to **pain or training/management issues**, and that linking behaviour to hormones is often a diagnostic dilemma (Crabtree, 2020; Carroll et al., 2023; Hothersall & Casey, 2012).

**Sex, ridden behaviour and welfare**

- A large survey (E‑BARQ) found **no significant sex‑related differences in ridden behaviour** between mares and geldings, challenging the idea that mares are generally more difficult under saddle (Aune et al., 2020).
- Broader welfare work links ridden “hyperreactive” behaviour mainly to welfare and management factors, not to sex or presumed hormonal effects (Luke et al., 2021; O’Connell et al., 2025; Bell et al., 2019; Hothersall & Casey, 2012).

**Conclusion**

Current evidence does **not** provide a clear, experimental link between specific oestrous/ovulatory stages or measured hormone levels and the *amount* of unwanted behaviour when mares are ridden. Studies instead emphasise: (1) substantial normal variation over the cycle, (2) frequent over‑attribution of ridden problems to ovaries/hormones, and (3) the importance of pain, training history, and welfare in explaining “moody mare” behaviour.

*These search results were found and analyzed using Consensus, an AI-powered search engine for research. Try it at* [*https://consensus.app*](https://consensus.app/)*. © 2026 Consensus NLP, Inc. Personal, non-commercial use only; redistribution requires copyright holders’ consent.*

**References**

Melgaard, D., Korsgaard, T., Thoefner, M., Petersen, M., & Pedersen, H. (2020). Moody Mares—Is Ovariectomy a Solution?. *Animals : an Open Access Journal from MDPI*, 10. <https://doi.org/10.3390/ani10071210>

Crabtree, J. (2020). A review of oestrus suppression techniques in mares. *Equine Veterinary Education*. <https://doi.org/10.1111/eve.13405>

Huggins, L., Norris, J., Conley, A., & Dini, P. (2023). Abnormal mare behaviour is rarely associated with changes in hormonal markers of granulosa cell tumours: A retrospective study.. *Equine veterinary journal*. <https://doi.org/10.1111/evj.13967>

Stachurska, A., Kędzierski, W., Kaczmarek, B., Wiśniewska, A., Żylińska, B., & Janczarek, I. (2023). Variation of Physiological and Behavioural Parameters during the Oestrous Cycle in Mares. *Animals : an Open Access Journal from MDPI*, 13. <https://doi.org/10.3390/ani13020211>

Crowell‐Davis, S. (2007). Sexual behavior of mares. *Hormones and Behavior*, 52, 12-17. <https://doi.org/10.1016/j.yhbeh.2007.03.020>

Aune, A., Fenner, K., Wilson, B., Cameron, E., McLean, A., & McGreevy, P. (2020). Reported Behavioural Differences between Geldings and Mares Challenge Sex-Driven Stereotypes in Ridden Equine Behaviour. *Animals : an Open Access Journal from MDPI*, 10. <https://doi.org/10.3390/ani10030414>

Straticò, P., Hattab, J., Guerri, G., Carluccio, A., Bandera, L., Celani, G., Marruchella, G., Varasano, V., & Petrizzi, L. (2023). Behavioral Disorders in Mares with Ovarian Disorders, Outcome after Laparoscopic Ovariectomy: A Case Series. *Veterinary Sciences*, 10. <https://doi.org/10.3390/vetsci10080483>

Carroll, S., Sykes, B., & Mills, P. (2023). Understanding and treating equine behavioural problems.. *Veterinary journal*, 105985. <https://doi.org/10.1016/j.tvjl.2023.105985>

O’Connell, E., Dyson, S., McLean, A., & McGreevy, P. (2025). No More Evasion: Redefining Conflict Behaviour in Human–Horse Interactions. *Animals : an Open Access Journal from MDPI*, 15. <https://doi.org/10.3390/ani15030399>

Bell, C., Rogers, S., Taylor, J., & Busby, D. (2019). Improving the Recognition of Equine Affective States. *Animals : an Open Access Journal from MDPI*, 9. <https://doi.org/10.3390/ani9121124>

Asa, C., Goldfoot, D., & Ginther, O. (1979). Sociosexual behavior and the ovulatory cycle of ponies (Equus caballus) observed in harem groups. *Hormones and Behavior*, 13, 49-65. <https://doi.org/10.1016/0018-506x(79)90034-5>

Hedberg, Y., Dalin, A., Ohagen, P., Kr, H., & Kindahl, H. (2005). Effect of oestrous-cycle stage on the response of mares in a novel object test and isolation test.. *Reproduction in domestic animals = Zuchthygiene*, 40 5, 480-8. <https://doi.org/10.1111/j.1439-0531.2005.00611.x>

Luke, K., McAdie, T., Smith, B., & Warren-Smith, A. (2021). New insights into ridden horse behaviour, horse welfare and horse-related safety. *Applied Animal Behaviour Science*. <https://doi.org/10.1016/j.applanim.2021.105539>

Asa, C., & Asa, C. (1986). Sexual behavior of mares.. *The Veterinary clinics of North America. Equine practice*, 2 3, 519-34. <https://doi.org/10.1016/s0749-0739(17)30704-6>

Hothersall, B., & Casey, R. (2012). Undesired behaviour in horses: A review of their development, prevention, management and association with welfare. *Equine Veterinary Education*, 24, 479-485. <https://doi.org/10.1111/j.2042-3292.2011.00296.x>

Prompt 2: 08.11.2025

Regarding this statement: “Human sociability was most associated with age, with scores decreasing with horse age and stabilising after about 20 years old. This is in line with behavioural observations from Lerch et al. (2021), where, in a motionless person test, 3–15-year-old equids interacted more with the human than those >15 years, suggesting a non-linear age effect with reduced interaction in the oldest individuals.” Can you find me similar results in other species? Are there publications that show results going against these findings in horses, or that investigated horse age and human sociability as a personality factor and did not find any correlation?

Output:

**Age and Human-Directed Sociability in Horses and Other Species**

**Age–sociability patterns in other species**

Across mammals, **social engagement often declines or becomes more selective with age**, giving a pattern broadly similar to the reduced human‑directed sociability you cite in older horses.

- In humans, trait sociability scores decline with age and are linked to age‑related changes in intrinsic functional brain networks (Dan et al., 2025).
- Reviews on social ageing in mammals and primates report smaller social networks and greater selectivity with age in several species (Machanda & Rosati, 2020; Siracusa et al., 2022; Zhang et al., 2023; Siracusa et al., 2022).
- Long‑term data in rhesus macaques show that older females reduce the number of social partners while maintaining similar total time spent socializing, i.e., **more selective but not less socially motivated** (Siracusa et al., 2022).
- Tibetan macaque females approach conspecifics less and experience fewer attacks as they age, consistent with conflict avoidance and reduced social initiation in older individuals (Zhang et al., 2023).

These findings support a general, though not universal, trend toward **narrower or reduced social engagement in late life** across mammals.

**Example overview**

| **Species/group** | **Age trend in sociality** | **Citations** |
| --- | --- | --- |
| Humans | Sociability scores decline with age | (Dan et al., 2025; Machanda & Rosati, 2020) |
| Rhesus macaques | Fewer partners, stable social time (selectivity) | (Siracusa et al., 2022) |
| Tibetan macaques | Older females initiate fewer approaches | (Zhang et al., 2023) |

**Figure 1:** Age-related shifts in sociality across species.

**Evidence in horses: support vs potential contradictions**

Most work on horses addresses human–horse interaction broadly, not age–sociability as a personality trait per se.

- A recent study on following human cues found **better performance with increasing age** in a two‑way choice task, indicating improved socio‑cognitive use of human signals, not reduced engagement (Liehrmann et al., 2023).
- Neuroendocrine work found **higher oxytocin in aged horses** and a positive correlation between serotonin and friendliness to humans, but did not report a simple negative linear age–friendliness relationship (Kim & Yoon, 2022).
- Reviews of horse–human interaction emphasize that approach vs avoidance and affiliative responses are shaped strongly by training history, management, pain, and individual temperament, which can obscure simple age effects (Hall & Kay, 2024; Borda et al., 2023).
- Personality studies show traits like excitability/anxiousness predicting less human‑directed behaviour, but age is not highlighted as the main driver (Henriksson et al., 2019; Schrimpf et al., 2020; Borda et al., 2023).

Within this set of papers, **no clear publication directly contradicts Lerch et al.’s non‑linear age pattern by showing “no correlation” between age and human sociability as a personality dimension**, but several results (e.g., improved cue‑use with age (Liehrmann et al., 2023), hormone patterns (Kim & Yoon, 2022)) suggest that age effects can be task‑ or mechanism‑specific rather than uniformly negative.

**Conclusion**

Across humans and other primates, ageing is often associated with reduced or more selective sociality, broadly compatible with reduced human‑directed interaction in the oldest horses. In horses, existing work points to complex age effects (e.g., better cue‑following with age, hormone changes) rather than a simple monotonic decline in friendliness, and specific “no‑effect” or contrary findings on age vs human‑sociability as a personality trait are not clearly documented in the retrieved literature.

*These search results were found and analyzed using Consensus, an AI-powered search engine for research. Try it at* [*https://consensus.app*](https://consensus.app/)*. © 2026 Consensus NLP, Inc. Personal, non-commercial use only; redistribution requires copyright holders’ consent.*

**References**

Machanda, Z., & Rosati, A. (2020). Shifting sociality during primate ageing. *Philosophical Transactions of the Royal Society B*, 375. <https://doi.org/10.1098/rstb.2019.0620>

Siracusa, E., Valle, J., Phillips, D., Platt, M., Higham, J., Snyder‐Mackler, N., & Brent, L. (2022). Within-individual changes reveal increasing social selectivity with age in rhesus macaques. *Proceedings of the National Academy of Sciences of the United States of America*, 119. <https://doi.org/10.1101/2022.05.31.494118>

Kim, J., & Yoon, M. (2022). The Effect of Serotonin and Oxytocin on Equine Docility and Friendliness to Humans. *Journal of Veterinary Behavior*. <https://doi.org/10.1016/j.jveb.2022.01.004>

Liehrmann, O., Cosnard, C., Riihonen, V., Viitanen, A., Alander, E., Jardat, P., Koski, S., Lummaa, V., & Lansade, L. (2023). What drives horse success at following human-given cues? An investigation of handler familiarity and living conditions. *Animal Cognition*, 26, 1283 - 1294. <https://doi.org/10.1007/s10071-023-01775-0>

Zhang, T., Liu, S., Xia, Y., Li, B., Wang, X., & Li, J. (2023). Aging-Related Behavioral Patterns in Tibetan Macaques. *Biology*, 12. <https://doi.org/10.3390/biology12101325>

Henriksson, J., Sauveroche, M., & Roth, L. (2019). Effects of size and personality on social learning and human-directed behaviour in horses (Equus caballus). *Animal Cognition*, 22, 1001 - 1011. <https://doi.org/10.1007/s10071-019-01291-0>

Dan, Y., Siew, S., & Yu, J. (2025). Intrinsic functional connectivity brain networks mediate effect of age on sociability. *PLOS One*, 20. <https://doi.org/10.1371/journal.pone.0324277>

Siracusa, E., Higham, J., Snyder‐Mackler, N., & Brent, L. (2022). Social ageing: exploring the drivers of late-life changes in social behaviour in mammals. *Biology Letters*, 18. <https://doi.org/10.1098/rsbl.2021.0643>

Borda, T., Auer, U., & Jenner, F. (2023). Equine Social Behaviour: Love, War and Tolerance. *Animals : an Open Access Journal from MDPI*, 13. <https://doi.org/10.3390/ani13091473>

Schrimpf, A., Single, M., & Nawroth, C. (2020). Social Referencing in the Domestic Horse. *Animals : an Open Access Journal from MDPI*, 10. <https://doi.org/10.3390/ani10010164>

Hall, C., & Kay, R. (2024). Living the good life? A systematic review of behavioural signs of affective state in the domestic horse (Equus caballus) and factors relating to quality of life. Part 2: Horse-human interactions. *Animal Welfare*, 33. <https://doi.org/10.1017/awf.2024.41>

Prompt 3: 13.11.2025

Can you find me sources for horse attentional state and learning abilities in relation to age?

**Key sources on age, attention, and learning in horses**

Below are primary research papers directly linking **age** with **attentional state** and/or **learning/memory** in horses, plus closely related work you can cite.

**Core age–learning/memory studies in horses**

| **Focus** | **Main finding relevant to age** | **Citations** |
| --- | --- | --- |
| Short- and long‑term memory in adult vs senior horses | Adult (5–15 y) and senior (≥16 y) horses both learned a target‑touch task and retained it for 10 days, but seniors took significantly longer to complete the task at retest, indicating **slower retrieval rather than loss of associative learning** (Cellai et al., 2024) | (Cellai et al., 2024) |
| Discrimination learning: age effects | In a visual three‑choice discrimination task, older horses showed **slower learning**; age was **negatively correlated** with rate of learning (Mader & Price, 1980) | (Mader & Price, 1980) |
| Social learning: age, rank, exploration | In a feeding‑apparatus task, **young, low‑ranking, more exploratory horses learned more readily by observing older group members**, while older horses learned more slowly, suggesting age‑related differences in social learning strategies (Krueger et al., 2014) | (Krueger et al., 2014) |
| Age, learning & early handling | Quarter Horse weanlings given more handling up to 18 months later showed **better maze learning at 2 years** and higher trainability ratings; the least‑handled group made fewer correct responses, linking early experience, emotionality, and later learning ability (Heird et al., 1986) | (Heird et al., 1986) |
| Learning/memorisation in two tasks | Classic paper on **age, sex, sire** effects on learning and memory in two tasks (Applied Animal Behaviour Science); widely cited for age effects, though the abstract text is not available here (Wolff & Hausberger, 1996) | (Wolff & Hausberger, 1996) |
| Learning performance at 1 vs 2 years | Young Warmbloods tested at 1 and 2 years showed **stable individual learning performance over time** in avoidance learning, indicating consistent cognitive traits across early ages (Visser et al., 2003) | (Visser et al., 2003) |

**Figure 1:** Key experimental sources on age and equine learning

**Age, attentional state, and socio‑cognitive skills**

| **Aspect** | **Age-related insight** | **Citations** |
| --- | --- | --- |
| Attentional characteristics & work type | In a visual attention test, **age did not significantly affect attentional pattern**, whereas type of work (e.g. riding school vs sport) strongly shaped attention (sequence length, fragmentation) (Rochais et al., 2022) | (Rochais et al., 2022) |
| Following human cues: age | In a two‑way choice task using human pointing/gaze, **success increased with age**, independent of handler familiarity; living in groups and on pasture also improved performance, linking age and environment to socio‑cognitive skill toward humans (Liehrmann et al., 2023) | (Liehrmann et al., 2023) |
| Development of sensitivity to human attention | Horses <3 years could use coarse cues (body orientation) but not subtle ones (head/eye cues) to judge human attention; full sensitivity appears to require **experience and age**, while object-choice cue use is present earlier (Proops et al., 2013) | (Proops et al., 2013) |
| Age, boldness, independence | Large owner‑survey data showed **older horses rated as bolder**, a trait related to attention to novel stimuli and potentially to learning context, though independence did not increase with age (Burattini et al., 2020) | (Burattini et al., 2020) |

**Figure 2:** Studies linking age with attention-like and socio-cognitive traits

**Additional potentially useful sources**

- **Exploratory behaviour and learning in young horses** – Object exploration (a proxy for curiosity/attentional engagement) at 5 months and 1 year predicted better performance in both visual discrimination and pressure‑release learning tasks, independent of fearfulness (Christensen et al., 2021).
- **Personality and habit formation** – Fearful horses were more prone to form **habitual** rather than goal‑directed responses in an instrumental learning task; while not age‑focused, this is relevant to attentional control and learning style (Lansade et al., 2017).
- **Innovative problem‑solving** – Age included as an individual factor in innovative feeder task; sample small, but provides data on age, stress hormones, lateralization, and problem‑solving style (Esch et al., 2019).

These papers together give empirical coverage of how age relates to equine learning speed, memory retention, social learning, use of human cues, and some aspects of attentional style.

*These search results were found and analyzed using Consensus, an AI-powered search engine for research. Try it at* [*https://consensus.app*](https://consensus.app/)*. © 2026 Consensus NLP, Inc. Personal, non-commercial use only; redistribution requires copyright holders’ consent.*

**References**

Cellai, S., Gazzano, A., Casini, L., Gazzano, V., Cecchi, F., Macchioni, F., Cozzi, A., Pageat, L., Arroub, S., Fratini, S., Felici, M., Curadi, M., & Baragli, P. (2024). The Memory Abilities of the Elderly Horse. *Animals : an Open Access Journal from MDPI*, 14. <https://doi.org/10.3390/ani14213073>

Christensen, J., Ahrendt, L., Malmkvist, J., & Nicol, C. (2021). Exploratory behaviour towards novel objects is associated with enhanced learning in young horses. *Scientific Reports*, 11. <https://doi.org/10.1038/s41598-020-80833-w>

Rochais, C., Stomp, M., Sébilleau, M., Houdebine, M., Henry, S., & Hausberger, M. (2022). Horses’ attentional characteristics differ according to the type of work. *PLoS ONE*, 17. <https://doi.org/10.1371/journal.pone.0269974>

Proops, L., Rayner, J., Taylor, A., & McComb, K. (2013). The Responses of Young Domestic Horses to Human-Given Cues. *PLoS ONE*, 8. <https://doi.org/10.1371/journal.pone.0067000>

Liehrmann, O., Cosnard, C., Riihonen, V., Viitanen, A., Alander, E., Jardat, P., Koski, S., Lummaa, V., & Lansade, L. (2023). What drives horse success at following human-given cues? An investigation of handler familiarity and living conditions. *Animal Cognition*, 26, 1283 - 1294. <https://doi.org/10.1007/s10071-023-01775-0>

Esch, L., Wöhr, C., Erhard, M., & Krüger, K. (2019). Horses’ (Equus Caballus) Laterality, Stress Hormones, and Task Related Behavior in Innovative Problem-Solving. *Animals : an Open Access Journal from MDPI*, 9. <https://doi.org/10.3390/ani9050265>

Mader, D., & Price, E. (1980). Discrimination learning in horses: effects of breed, age and social dominance.. *Journal of animal science*, 50 5, 962-5. <https://doi.org/10.2527/jas1980.505962x>

Burattini, B., Fenner, K., Anzulewicz, A., Romness, N., McKenzie, J., Wilson, B., & McGreevy, P. (2020). Age-Related Changes in the Behaviour of Domestic Horses as Reported by Owners. *Animals : an Open Access Journal from MDPI*, 10. <https://doi.org/10.3390/ani10122321>

Krueger, K., Farmer, K., & Heinze, J. (2014). The effects of age, rank and neophobia on social learning in horses. *Animal Cognition*, 17, 645-655. <https://doi.org/10.1007/s10071-013-0696-x>

Lansade, L., Marchand, A., Coutureau, E., Ballé, C., Polli, F., & Calandreau, L. (2017). Personality and predisposition to form habit behaviours during instrumental conditioning in horses (Equus caballus). *PLoS ONE*, 12. <https://doi.org/10.1371/journal.pone.0171010>

Wolff, A., & Hausberger, M. (1996). Learning and memorisation of two different tasks in horses: the effects of age, sex and sire. *Applied Animal Behaviour Science*, 46, 137-143. <https://doi.org/10.1016/0168-1591(95)00659-1>

Heird, J., Whitaker, D., Bell, R., Ramsey, C., & Lokey, C. (1986). THE EFFECTS OF HANDLING AT DIFFERENT AGES ON THE SUBSEQUENT LEARNING ABILITY OF 2-YEAR-OLD HORSES. *Applied Animal Behaviour Science*, 15, 15-25. <https://doi.org/10.1016/0168-1591(86)90018-3>

Visser, E., Reenen, C., Schilder, M., Barneveld, A., & Blokhuis, H. (2003). Learning performances in young horses using two different learning tests.. *Applied Animal Behaviour Science*, 80, 311-326. <https://doi.org/10.1016/s0168-1591(02)00235-6>

Prompt 4: 13.11.2025

What are the sources regarding the use of a bit as a potential source of pain and discomfort in horses? What are the sources going against this idea?

Output :

**Bits as Sources of Pain in Horses: Supporting and Opposing Evidence**

Most current research indicates that **bits commonly cause oral pain, lesions, and conflict behaviour**, although severity depends on bit design, fit, rein use, and management.

**Evidence That Bits Cause Pain/Discomfort**

- **Physiology & behaviour:** Oral tissues (bars, tongue, lips, buccal mucosa) are highly innervated and sensitive; compression, laceration, ischemia, and stretching from bits are all noxious stimuli, leading to pain, anxiety and fear, plus secondary breathlessness in some head–neck positions (Mellor, 2020; Seck et al., 2022; Anttila et al., 2022).
- **Lesions in competition horses:**
  - Eventers: 52% had acute bit-area lesions post–cross-country; 30% moderate/severe. Thin (10–13 mm) and thick (18–22 mm) bits carried higher risk than mid‑thickness bits (Tuomola et al., 2021).
  - Finnish trotters: specific bit types (Crescendo, mullen mouth regulator, straight plastic) had much higher odds of moderate–severe lesions; however, lesions occurred with all bit types (Tuomola et al., 2020).
  - Danish competition horses: visible lip commissure abrasions/blood in 9.2%; risk increased with tighter nosebands, showing interaction between bit and tack pressure (Uldahl & Clayton, 2019).
- **Working horses:** In Senegal, replacing crude iron bits with improved designs significantly reduced lip, tongue, buccal and bar lesions and reduced open‑mouth, tongue‑lolling and head‑shaking behaviours, without loss of control (Seck et al., 2022).
- **Fit and misfit:** Many horses are bitted with inappropriate thickness/length; misfit bits compress the tongue or bars and can cause trauma and evasion behaviours (mouth opening, protruding tongue, grabbing the bit) (Anttila et al., 2022).
- **Pain ethology:** Reviews and ethograms of discomfort identify bit‑related oral injury and oral behaviours (mouth opening, head tossing, tongue extension) as probable pain indicators (Mellor, 2020; Seck et al., 2022; Torcivia & McDonnell, 2021; Anttila et al., 2022).

**Illustrative Summary of Bit-Related Problems**

| **Aspect** | **Main finding** | **Citations** |
| --- | --- | --- |
| Acute mouth lesions in sport horses | 30–56% moderate/severe in some samples | (Tuomola et al., 2021; Tuomola et al., 2020; Uldahl & Clayton, 2019) |
| Behavioural signs (open mouth, head toss, tongue out) | Linked to evasion of bit pressure and oral discomfort | (Mellor, 2020; Seck et al., 2022; Dyson & Pollard, 2024; Torcivia & McDonnell, 2021; Anttila et al., 2022) |
| Welfare improvements with better/bitless designs | New bits or bit‑free bridles reduce lesions and aversive behaviours | (Mellor, 2020; Seck et al., 2022; Fretheim-Kelly et al., 2020; Anttila et al., 2022) |

**Figure 1:** Summary of key documented bit-related welfare issues.

**Evidence Against or Limiting the Bit–Pain Link**

- **Some horses show few visible lesions:** Around half of eventers had no acute visible lesions after competition (Tuomola et al., 2021), and many Danish competition horses had normal commissures (Uldahl & Clayton, 2019).
- **Respiratory effects not always bit‑dependent:** In horses with dynamic laryngeal collapse, switching from a snaffle to a bitless bridle did **not** reduce the severity of laryngeal collapse; head–neck angle and rein tension, not the bit itself, drove the problem (Fretheim-Kelly et al., 2020).
- **Multifactorial causation:** Repeated mouth opening or conflict behaviours can also reflect dental disease, overly tight nosebands, poor riding, or other pain sources, not just the bit (Kennedy, 2025; Laukkanen et al., 2022; Grauw & Loon, 2016).
- **Control and safety arguments:** Some authors and regulatory frameworks still regard bits as necessary for precise control, with concerns (rather than data) that bitless options may be unsafe in some contexts (Mellor, 2020; Tuomola et al., 2020).

**Conclusion**

Overall, peer‑reviewed evidence strongly supports bits as **common contributors** to oral injury, pain and conflict behaviour, modulated by bit type, fit, rein use and concurrent tack. Evidence “against” the idea mainly shows that not all problems are bit‑caused and that some conditions (e.g., specific airway disorders) are driven more by head–neck posture than by the bit itself.

*These search results were found and analyzed using Consensus, an AI-powered search engine for research. Try it at* [*https://consensus.app*](https://consensus.app/)*. © 2026 Consensus NLP, Inc. Personal, non-commercial use only; redistribution requires copyright holders’ consent.*

**References**

Mellor, D. (2020). Mouth Pain in Horses: Physiological Foundations, Behavioural Indices, Welfare Implications, and a Suggested Solution. *Animals : an Open Access Journal from MDPI*, 10. <https://doi.org/10.3390/ani10040572>

Tuomola, K., Mäki-Kihniä, N., Valros, A., Mykkänen, A., & Kujala-Wirth, M. (2021). Bit-Related Lesions in Event Horses After a Cross-Country Test. *Frontiers in Veterinary Science*, 8. <https://doi.org/10.3389/fvets.2021.651160>

Seck, M., Jobling, R., & Brown, A. (2022). Trialling Locally Made, Low-Cost Bits to Improve Bit-Related Welfare Problems in Cart Horses: Findings from a Study in Senegal. *Animals : an Open Access Journal from MDPI*, 13. <https://doi.org/10.3390/ani13010002>

Tuomola, K., Mäki-Kihniä, N., Valros, A., Mykkänen, A., & Kujala-Wirth, M. (2020). Risk factors for bit‐related lesions in Finnish trotting horses. *Equine Veterinary Journal*, 53, 1132 - 1140. <https://doi.org/10.1111/evj.13401>

Kennedy, R. (2025). The challenge of assessing dental pain in horses.. *The Veterinary record*, 196 3, 111-113. <https://doi.org/10.1002/vetr.5188>

Dyson, S., & Pollard, D. (2024). Determination of Equine Behaviour in Subjectively Non-Lame Ridden Sports Horses and Comparison with Lame Sports Horses Evaluated at Competitions. *Animals : an Open Access Journal from MDPI*, 14. <https://doi.org/10.3390/ani14121831>

Laukkanen, T., Karma, L., Virtala, A., Mykkänen, A., Pehkonen, J., Rossi, H., Tuomola, K., & Raekallio, M. (2022). Behavioural Signs Associated with Equine Cheek Tooth Findings.. *Journal of equine veterinary science*, 104198. <https://doi.org/10.2139/ssrn.4212254>

Grauw, J., & Loon, J. (2016). Systematic pain assessment in horses.. *Veterinary journal*, 209, 14-22. <https://doi.org/10.1016/j.tvjl.2015.07.030>

Torcivia, C., & McDonnell, S. (2021). Equine Discomfort Ethogram. *Animals : an Open Access Journal from MDPI*, 11. <https://doi.org/10.3390/ani11020580>

Uldahl, M., & Clayton, H. (2019). Lesions associated with the use of bits, nosebands, spurs and whips in Danish competition horses. *Equine Veterinary Journal*, 51, 154–162. <https://doi.org/10.1111/evj.12827>

Fretheim-Kelly, Z., Fjordbakk, C., Fintl, C., Krontveit, R., & Strand, E. (2020). A bitless bridle does not limit or prevent Dynamic Laryngeal Collapse.. *Equine veterinary journal*. <https://doi.org/10.1111/evj.13287>

Anttila, M., Raekallio, M., & Valros, A. (2022). Oral Dimensions Related to Bit Size in Adult Horses and Ponies. *Frontiers in Veterinary Science*, 9. <https://doi.org/10.3389/fvets.2022.879048>
